# Tribological Response of Glass Fiber/Polyester Composites After Pressurized Water-Immersion Aging Assessed by Reciprocating and Ball-on-Disc Wear Testing

**DOI:** 10.3390/polym17182503

**Published:** 2025-09-17

**Authors:** Sinan Fidan, Satılmış Ürgün, Mehmet İskender Özsoy, Mustafa Özgür Bora, Erman Güleç

**Affiliations:** 1 Department of Airframe & Powerplant Maintenance, Faculty of Aeronautics and Astronautics, Kocaeli University, Kocaeli 41001, Türkiye; sfidan@kocaeli.edu.tr (S.F.); ozgur.bora@kocaeli.edu.tr (M.Ö.B.); 2 Department of Aviation Electrics and Electronics, Faculty of Aeronautics and Astronautics, Kocaeli University, Kocaeli 41001, Türkiye; urgun@kocaeli.edu.tr; 3 Department of Mechanical Engineering, Faculty of Engineering, Sakarya University, Sakarya 54050, Türkiye; 4 Otokar Automotive Defense Industry Corp., Sakarya 54580, Türkiye; egulec@otokar.com.tr

**Keywords:** glass fiber-reinforced polymer laminates, pressurized water aging, ball-on-disc tribology, reciprocating sliding wear, coefficient of friction

## Abstract

This study quantifies how pressurized water-immersion aging degrades the tribological response of cross-ply E-glass/polyester laminates by coupling dual-mode testing with surface metrology and factorial ANOVA. Eleven-ply [0/90]s plates were aged at 10 bar for 0, 7, 14, and 21 days, gaining 10% mass (72.2 to 79.4 g), then tested under 20 N in ball-on-disc (50–100 mm s^−1^; 100–200 m) and reciprocating modes (1–2 Hz; 10–20 m). In ball-on-disc tests, steady-state COF rose from 0.40 to 0.47 (unaged) to 0.49 to 0.52 (14–21 days), and the low-friction run-in largely vanished with aging. Wear scar width and depth increased from 1.38 to 1.90 mm and 75 to 117 µm, respectively. Reciprocating tests showed a non-monotonic trend: moderate aging lowered COF to 0.50, whereas 21 days produced the harshest response (up to 0.78) and the widest/deepest scars (1.15 to 1.95 mm; 40 to 110 µm). ANOVA revealed that, in ball-on-disc tests, the COF was governed by sliding distance (28.70%) and speed (24.64%), with a strong Days × Speed interaction (31.66%); track-depth variance was dominated by distance (42.16%) and aging (32.16%). For the COF under reciprocating tests, aging was the leading main effect (21.21%), with large Days × Frequency (20.36%) and Days × Track (20.03%) interactions. Uniquely, this study isolates the effect of controlled hydrostatic aging (10 bar) and compares two sliding kinematics under identical loads, establishing quantitative thresholds (14 and 21 days) where interfacial debonding and third-body abrasion accelerate.

## 1. Introduction

Fiber-reinforced polymer composites (FRPCs) have become ubiquitous materials in structural, aerospace, automotive, and biomedical applications due to their high specific strength, light weight, and tunable tribological performance [1,2,3,4,5,6,7,8,9,10]. Nonetheless, despite their positive attributes, environmental conditions like thermal, hygrothermal, and hydrostatic aging manage to considerably compromise their mechanical performance and wear behavior, especially if moisture-induced deterioration compromises the fiber–matrix interface. Extensive scientific research has thoroughly investigated the influence of aging and environmental exposure on synthetic and natural fiber-reinforced composites’ tribological behavior. Autay et al. [11] researched short glass fiber-reinforced polyamide 66 (PA66) composites that were exposed to accelerated thermal aging regimes. Findings showed that flexural strength was decreased and tribological behavior was altered with increasing thermal aging time, especially in composites that contained high fiber contents, which showed that high temperatures magnify interfacial adhesion weakening and high wear rates. Chaudhary and Ahmad [12] broadened this focus further, considering natural fiber epoxy composites, that is, jute, hemp, flax, and their respective blends, after prolonged water-immersion aging. Findings showed extreme dynamic mechanical property transitions, wherein storage and loss modulus increased by up to 763% and 590%, respectively, after aging, which tribologically corresponded to elevated friction coefficients and enhanced wear rates, evidencing extreme water absorption-induced deterioration in the mechanical performance of natural-fiber composites. In an even broader study, Bandaru et al. [13] examined hygrothermal aging of water-immersed glass/PTFE hybrid composites. Much like Chaudhary and Ahmad, significantly reduced abrasive wear resistance was observed, especially after 60 days of exposure at 35 °C, which emphasized how, even in hybrid composites where PTFE would be assumed to facilitate lubrication, concurrent water and temperature exposure suppressed tribological behavior. At a microscale level, Liao et al. [14] investigated glass fiber epoxy composites aged under thermal exposure in air, simulated produced water, and oil environments. Nanoindentation and SEM assessments revealed widespread interfacial failures, where the interfacial modulus was increased up to 15.88 GPa in the near neighborhood of the fiber-reinforced matrix, yet interfacial strength decreased about 55% following hydrothermal exposure. Also, oxidative aging in air resulted in an 18% reduction in fiber strength, which emphasized matrix and fiber–matrix interface susceptibility to long-term thermal stresses. Additional information related to long-term durability was provided through Yekani Fard et al. [15], who investigated carbon fiber-reinforced polymer aged for two years and exposed to hygrothermal aging (60 °C and 90% relative humidity). Although resistance to crack growth was initially enhanced through fiber bridging mechanisms, overall interfacial integrity was lost, leading to unstable crack growth and deteriorated mechanical and tribological stability. Beyond aging-induced influences, composite formulations with multi-filler systems were investigated as an alternative to boost wear performance. Tian et al. [16] synthesized multi-filler epoxy composites for coated steel applications, reporting that, although friction coefficients were relatively stable (with 12% deviation) after subjection to hygrothermal aging, anti-wear performance was sacrificed, which was accounted for through matrix plasticization and deteriorated filler–matrix adhesion. Natural fiber-based composites have also attracted interest through their sustainability, yet investigations from Kumar and Bhowmik [17] regarding kenaf–pineapple laminated bio composites indicated that accelerated weathering and exposure to elevated humidity decreased surface integrity and tribological performance. Outcomes included enhanced roughness, hardness reduction, and extensive wear resistance deterioration upon mechanical loading, which were largely caused through moisture-induced swelling and interfacial debonding. In recent years, interest has turned towards optimizing fiber volume fractions. Birleanu et al. [18] studied glass fiber-reinforced polymer (GFRP) composites with different fiber fractions in terms of percentages of fiber weight fractions (50%, 65%, 70%) and observed that elevated fiber fractions caused reduced friction coefficients and wear rates. Through statistical analysis, their research indicated that fiber fractions contributed roughly 34% to variability in wear behavior, rendering it the key influencing factor, followed by loading and sliding velocity. Supporting research from the same research group [19] utilized statistical modeling approaches to further establish quantitative relationships between fiber content, contact temperature, and wear performance. Studies revealed that reinforcement with fibers assists in balancing out frictional temperatures through enhanced thermal dissipation, yet excessive loading elevates wear through amplified interaction on surfaces. Extending further towards biomedical applications, Mazur et al. [20] studied hygrothermal aging of high-density polyethylene (HDPE) composites reinforced with bio-fillers such as molybdenum disulfide (MoS_2_), cuttlebone, and red coral particles. The aged composites were found to exhibit improved tribological performance compared to pure HDPE, suggesting that well-selected fillers would be able to offset aging-induced wear loss. Sheng et al. [21] were able to manufacture dual-axis warp-knitted and plain-woven glass fiber/epoxy prepregs, which were hydrothermally aged for 800 h, 85 °C, and 100% RH. In their study, biaxial weld/thin prepreg warp-knitted composites, which were initially stronger (about 508 MPa tensile strength compared to about 465 MPa, and about 12% higher in bend strength), were seen to absorb a higher water content (6.10% compared to 5.50%), and lost more strength after aging (43.5% retention compared to 55.4% retention). Dynamic mechanical analysis showed that a higher loss of glass transition temperature occurred, and SEM showed that interfacial failure proceeded faster due to the open knit fabric structure, which would have been better defended against water penetration by an unwoven matrix like a plain-woven matrix. Yıldırım [22] studied glass fiber-reinforced polymer matrix composites embedded with SiC nanoparticles within 0–2 wt% that were aged, through artificial aging, at 70 °C and 85% RH for up to 1500 h. The researcher was able to predict the weight gained during aging accurately, reporting the mean squared error as only 0.001225—not only proving ANN-based forecasting feasible for moisture uptake in nanoparticle-reinforced GFRPs, but also establishing its high degree of feasibility. Haider et al. [23] studied silica fiber/epoxy radome composites subjected to accelerated environmental aging in an effort to determine their environmental performance in terms of their dielectric and thermal performance. Aging, according to their research, affected their dielectric constant, their dielectric loss, and their thermal conductivity—with emphasis now being laid on how selection of fiber–matrix architecture would be an important deciding factor in radome function retention during humid aging conditions. Meanwhile, in another study, Kini et al. [24] studied how hygrothermal aging affected nanoclay-filled silica/glass fiber/epoxy composites, optimizing responses through a full factorial design. Increasing nanoclay content, as well as an increase in glass fiber content, were seen to enhance mechanical behavior and offset aging according to their results, and their SEM analysis explicitly showed that higher reinforcement levels effectively minimized matrix-induced water damage. With Elium quadriaxial non-crimp glass fabric composites, Bandaru et al. [25] also showed that water aging for 60 days at 60 °C decreased tensile strength by ~30%, flexural strength by ~59%, interlaminar shear strength by ~46%, and Mode-II fracture toughness by ~37%, yet astonishingly increased Mode-I toughness by ~90%, which was attributed to observed SEM-based fiber bridging mechanisms. Aranha et al. [26] investigated hybrid polyester/glass/jute fiber composites with different stacking sequences produced via compression molding and VARTM, which were aged in water at 50 °C and 70 °C for 696 h. They showed that, independent of stacking sequence, tensile properties decreased following water exposure, and SEM showed delamination, fiber pull-out, fiber/matrix debonding, void formation, and resin removal. Kirar et al. [27] studied S2 woven glass fiber/epoxy laminates aged in artificial seawater for 4–12 months and subjected to quasistatic compression along different deformation rates. Aging decreased compressive strength by up to 23.5% and compressive modulus by up to 5.5%, with higher deformation rates accelerating performance loss through thickness and longitudinal directions. Yue et al. [28] carried out an integrated experimental and molecular dynamics study of CF/PEEK composite components subjected to hygrothermal environments. They elucidated that interfacial debonding is an overriding failure mode controlled by differential thermal expansion of CF and PEEK and enhanced water migration towards the matrix. In tribo-mechanical characterization, Khakbaz et al. [29] carried out an exhaustive study on glass fiber-reinforced polyamide 6 (PA6/GF) composites aged hydrothermally in water at 70 °C and 85% RH for 28 days. The coefficient of friction rose approximately 26.6%, and wear rate rose almost 29%, in line with extensive water uptake and matrix destruction corroborated through SEM micrograph images revealing interfacial debonding and crack nucleation. Şahin et al. [30] investigated carbon fiber-reinforced PEEK (CF/PEEK) composites subjected to hydrothermal exposure (80 °C, 85% RH), including samples that were subjected to thermal treatments. Results indicated that pre-aging heat treatment increases hydrothermal resistance through stabilization of the semi-crystalline PEEK matrix. Behera et al. [31] investigated SiC-reinforced short jute fiber/epoxy composites exposed to 60 °C and 95% RH conditions for 30 days of hydrothermal aging. Moisture-induced weight gain decreased from 4.87% for unreinforced composites to 2.95% for composites with SiC, suggesting improved resistance to hydrothermal degradation.

Collectively, these investigations confirm that synthetic and natural fiber-reinforced composites exhibit high environmental aging sensitivity, with moisture, temperature, and load being important degradation causes. Typically, deterioration progresses through interfacial weakening, matrix plasticization, micro-cracking, and high friction and wear rates. Nonetheless, strategic reinforcement fiber incorporation, filler use, and optimal fiber volume fractions can reverse some of these deleterious changes and improve the composites’ long-term tribological robustness. Based on the above literature, it is clear that, despite extensive investigations in earlier studies regarding hygrothermal and hydrothermal aging impacts on composite materials, considerable research gaps exist with respect to synergistic pressurized water-immersion aging and dual-mode tribological testing of cross-ply E-glass/polyester composites. Contrasted with typical studies that measure either natural immersion or stagnant-humidity environments, a controlled hydrostatic pressure aging process (10 bar) was utilized within this study to better replicate challenging service environments typical of marine and submerged applications. Operationally, the 10 bar hydrostatic level (1 MPa) approximates wet service conditions, e.g., the 100 m of seawater overhead encountered by marine/submerged structures and the pressurization of flooded composite housings/pipelines, thus providing a realistic driving force for water ingress and interface degradation without departing from the intended application envelope. E-glass/polyester laminate was selected to achieve a balance of mechanical performance, tribological behavior, and manufacturability for applications exposed to hydrostatic or aquatic environments. E-glass fibers provide high specific strength and stiffness, ensuring load-bearing capability while maintaining lightweight characteristics, which is critical for marine, submerged, and industrial components. The polyester matrix contributes adequate hydrolytic stability, limiting moisture-induced swelling, fiber–matrix debonding, and matrix plasticization, thereby maintaining tribological integrity under pressurized water immersion. Additionally, this research comprehensively combines both reciprocating and ball-on-disc wear evaluations within steady mechanical loading environments, allowing a direct intercomparison of wear behavior within two different sliding kinematics. Through extensive surface morphology measurements, coefficient of friction evolution tracking, and statistical factorial analysis, this research provides quantitative evidence of progressive interfacial fiber–matrix deterioration as a function of water exposure time. The research identifies important thresholds and characteristic transitions in wear mechanisms, advancing an in-depth comprehension of how pressurized water environments expedite tribological deterioration. Through careful correlation of aging time with wear track size, friction kinetics, and material loss, this research provides novel predictive monitors of GFRP laminate operational durability. Accordingly, this research fills an important gap within contemporary literature in explaining the synergistic impacts of pressure-assisted water aging as well as multi-conditional sliding kinematics, which provides valuable design guidelines for fiber-reinforced composites within challenging water environments.

## 2. Materials and Methods

### 2.1. Materials

The fiber used in our study was glass fiber with an area density of 600 g/m^2^ and fiber density of 2.550 g/cm^3^ (Metyx LT 600) from the supplier Metyx Composites (Istanbul, Türkiye). This fabric is made of a biaxial [0/90] structure, a standard count type with a 1200 tex yarn type and a 600 tex weft type. The resin used was Polipol 336-brand polyester resin (Istanbul, Türkiye) which has a density of 1.094 g/cm^3^ and a viscosity of 300 cP at room temperature according to the data of suppliers. The laminated composites consisted of a [0/90/0/90/0/90/0/90/0/90/0] stacking sequence. The laminate thickness was 5 mm and the fiber volume ratio (*V_f_*) was obtained as approximately 52%, theoretically, according to Equation (1).

(1) $$V_{f}=\frac{n.A_{w}}{\rho_{f}.h}$$

where *V_f_* is the fiber volume ratio (%), *n* is the layer number, *A_w_* is the fabric unit weight, *ρ_f_* is the fiber density, and *h* is the composite thickness [32].

**Figure 1:**
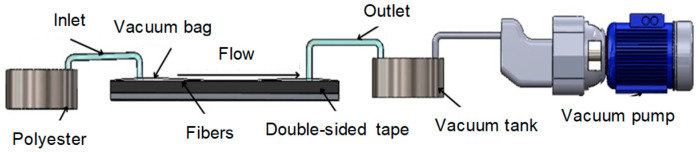
Schematic representation of the vacuum infusion process.

The vacuum infusion method was used for the manufacturing of composites, as shown in Figure 1. The following procedure was applied according to this manufacturing technique: (i) The glass fibers were laid up on the mold with the dimensions 200 mm × 200 mm. (ii) After the peel fabric and flow media were coated on the fibers, the vacuum bag was stretched with double-sided sealing tape. (iii) The vacuum motor was started and the impregnating of the polyester resin into the fibers was started. (iv) The process was completed when excess resin flowed into the vacuum tank. Then, the composite laminates were post-cured at 40 °C for 2 h. Test coupons were cut to have the dimensions 100 mm × 100 mm.

### 2.2. Experimental Setup

**Figure 2:**
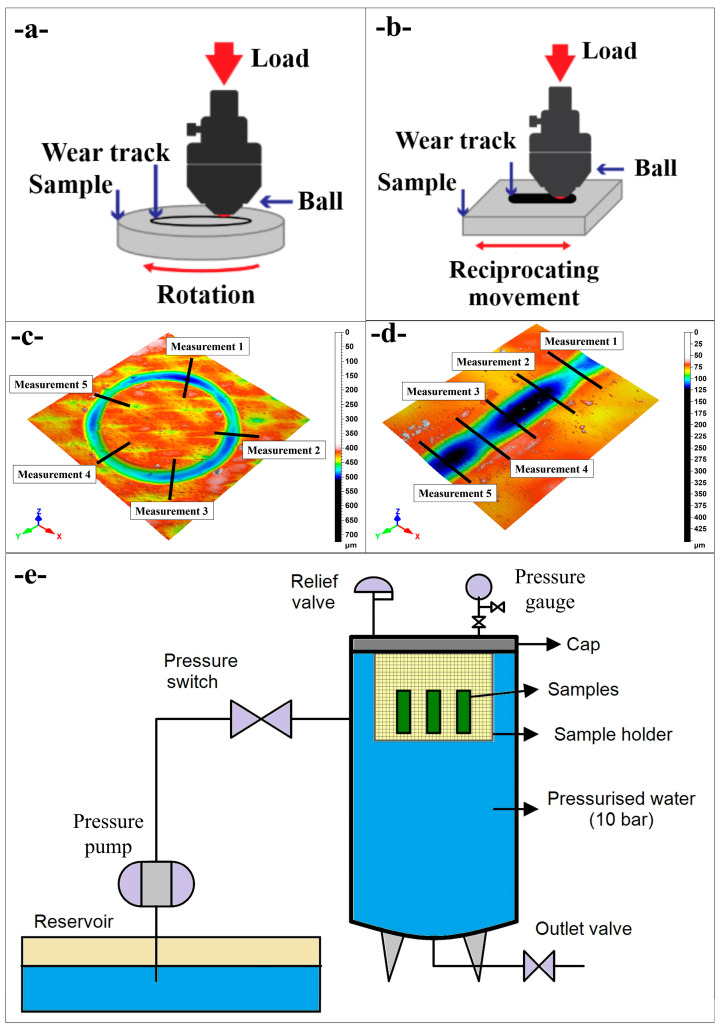
Experimental setup used in wear tests and water-immersion aging: (**a**) ball-on-disc wear test setup, (**b**) reciprocating wear test setup, (**c**) wear track measurements taken from ball-on-disc wear track, (**d**) wear track measurements taken from reciprocating wear track, (**e**) pressurized water aging test setup.

Figure 2a schematically illustrates the ball-on-disc configuration used on the UTS Tribolog™ Multi-Function Tribometer (Istanbul, Türkiye): a stationary 6 mm diameter counter-body (100Cr6 steel ball) was pressed against the horizontal, circular composite coupon under a constant normal load while the disc rotated beneath it, generating a concentric wear track whose diameter was fixed by the radial offset between the ball and the spindle axis. A 6 mm 100Cr6 steel ball (hardness 60–66 HRC; as-supplied surface finish Ra < 0.05 µm) slid against composite coupons with an initial surface roughness of Ra = 3 µm; before each run, both the counterface and specimen were ultrasonically cleaned in high-purity isopropanol and dried with oil-free nitrogen, and a brand-new ball was mounted for every test to avoid cross-contamination and pre-existing transfer films that could bias the early-distance COF and tribo-film nucleation. Figure 2b depicts the reciprocating mode of the same instrument, in which the ball remains fixed in the vertical axis but the flat specimen oscillates linearly under an identical load, producing an elongated wear scar whose length equals twice the stroke amplitude; stroke frequency and total sliding distance are controlled independently of the normal force, enabling direct comparison with the rotary tests. Unless otherwise stated, the COF time histories are raw (unfiltered) signals acquired at 1000 Hz; for visualization, traces were down-sampled to 10 Hz by non-overlapping 100-point boxcar averaging (reciprocating data additionally stroke-averaged per cycle), with no further filtering or baseline correction.

Figure 2c presents a representative three-dimensional surface map of a ball-on-disc scar acquired with the in situ white-light interferometer module. Five evenly spaced radial cross-sections (labeled Measurement 1 to 5) were extracted around the annulus to determine local scar width and depth; the mean of these five values was used in subsequent analyses, and the scatter served to calculate the percentage error bars reported in the results. Figure 2d shows the analogous topography for a reciprocating track. Here, five transverse profiles were taken perpendicular to the sliding direction at equidistant locations along the 20 mm stroke. Again, average width and depth and their associated uncertainties were derived from these replicate measurements, ensuring that wear metrics capture any longitudinal heterogeneity inherent to reciprocating motion.

Pressurized water-immersion aging was carried out in deionized water at 25 ± 1 °C, with the vessel maintained at 10.0 ± 0.1 bar; after depressurization, coupons were gently surface-dried and then conditioned for 24 h at 23 ± 2 °C and 50 ± 5% RH prior to tribological testing.

## 3. Results and Discussion

### 3.1. COF Analysis of Ball-on-Disc Wear Tests

**Figure 3:**
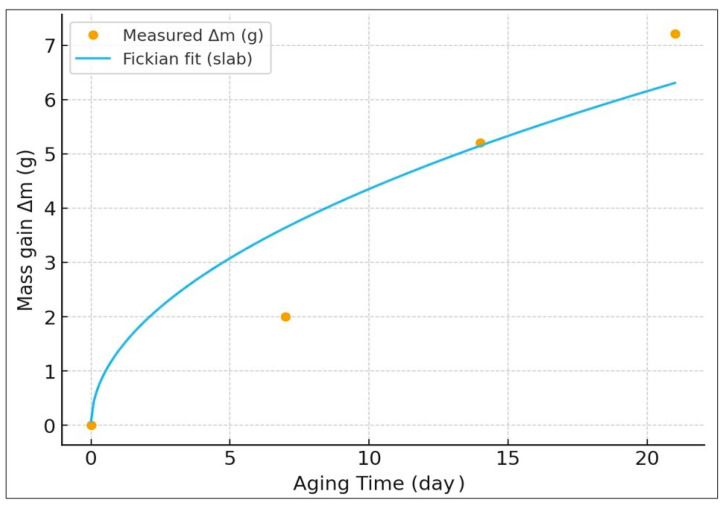
Mass gain vs. time curve (0, 7, 14, and 21 days) for the sample with 5 mm thickness.

Water absorption of the 5 mm thick specimen was studied, and the experimental data were modeled with the help of the Fickian diffusion model. Figure 3 shows the mass gain vs. time curve (0, 7, 14, and 21 days) for the sample with 5 mm thickness. The measured mass uptake showed a normal diffusion-control-type curve, that is, it showed fast increase in initial immersion periods and gradually moved towards equilibrium. The curve from experimental findings agreed well with the theoretical fitting, and it can be said that the absorptive mechanism can be well explained with Fick’s second law.

The calculated diffusion coefficient was found to be D = 2.1 × 10^−12^ m^2^/s, typical of a fairly slow moisture penetration rate of the polymer matrix. Such low values of diffusivity are normally associated with tight material structures and strong interfacial adhesions that slow down water molecule mobility. To avoid ambiguity, all diffusion parameters in Figure 3 are reported on a normalized basis: M(t) = Δm/m_0_, with a fitted saturation of M∞ = 0.10 g·g^−1^ (~10 wt%), which for the 100 × 100 × 5 mm coupon corresponds to an absolute mass gain of ~7.2 g (72.2 → 79.4 g); D = 2.1 × 10^−12^ m^2^·s^−1^ remains unchanged. These findings indicate that the diffusion process of the analyzed material is of a Fickian-controlled type, and the relatively low uptake at saturation suggests a satisfactory level of hydrothermal resistance. The simultaneous low levels of the diffusion coefficient and of the saturation level is of assistance for the achievement of long-term durability and for all those applications in which stability of dimension and resistance to degradation provoked by moisture are of primary importance.

**Figure 4:**
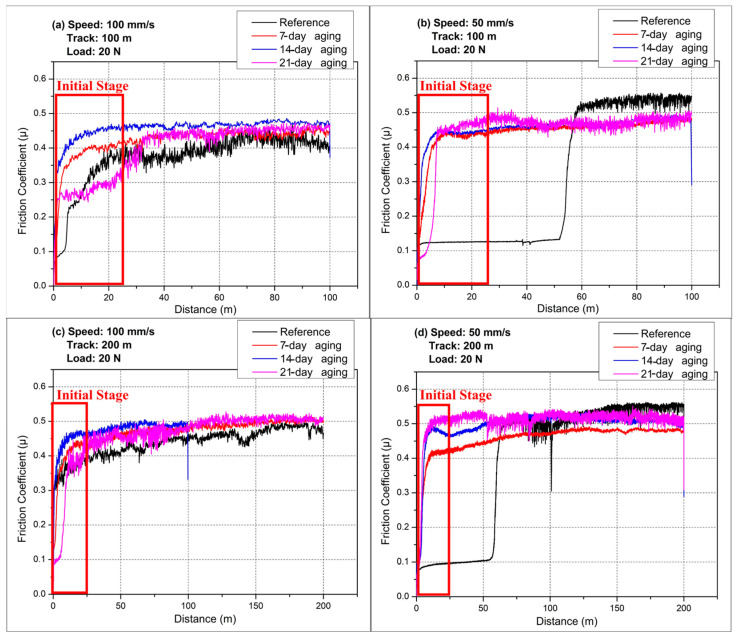
Effect of aging time on COF values at 20 N load in ball-on-disc wear tests: (**a**) 100 mm/s; 100 m; (**b**) 50 mm/s; 100 m; (**c**) 100 mm/s; 200 m; (**d**) 50 mm/s; 200 m. (The red rectangle shows the initial stage).

In Figure 4a, where the ball-on-disc test is run at 100 mm s^−1^ for a 100 m sliding distance, all specimens exhibit the classic two-stage evolution of the friction coefficient (µ): an extremely short run-in of <10 m followed by a quasi-steady regime. In Figure 4, the plateau friction is reported as the mean ± standard deviation (μ ± SD) over the steady-state window after excluding the running-in segment (<10 m for ball-on-disc). All red rectangle panels in the graphics share identical COF (–) y-limits and tick spacing; x-axes are harmonized by distance, and the early-window insets preserve the parent panel’s y-scale (no autoscaling) while zooming the marked regions. The reference laminate levels out at 0.40, whereas 7-day- and 14-day-aged specimens stabilize at 0.03–0.06 units higher, and the 21-day sample, despite starting slightly lower because of a lubricious resin film, converges to 0.45 by the end of the track. This progressive rise with aging is consistent with the measured water uptake (from 72.2 g for the virgin panel to 79.4 g after 21 days), which plasticizes the matrix and weakens the fiber–matrix interface, allowing larger debris to be entrained and increasing real contact area. Figure 4b lowers the sliding speed to 50 mm s^−1^ while keeping the track length at 100 m. The change in kinematics exposes a striking contrast between the unaged and aged laminates. The reference composite maintains a very low µ 0.10 for almost 70 m, implying the persistence of a resin-rich transfer film; once that film catastrophically fails, µ jumps abruptly to 0.52. In sharp distinction, all aged coupons reach their steady state within the first 5 m and remain in a narrow 0.44–0.48 band for the full distance. The longer residence time per contact event at 50 mm s^−1^ allows water-induced interface defects to dominate from the outset, precluding the formation of a stable lubricating layer and forcing direct glass–counterface interactions. Extending the track to 200 m at 100 mm s^−1^ in Figure 4c aggravates the cumulative damage. The reference sample experiences a gradual drift from 0.40 to 0.47 as micro-cracks multiply, whereas the 14-day- and 21-day-aged specimens oscillate around 0.49–0.52 almost from the beginning. A transient dip in the 21-day curve near 100 m suggests local delamination and momentary debris removal, but the coefficient quickly recovers, confirming that the degraded interface cannot sustain a protective tribo-layer over extended sliding. Finally, Figure 4d combines the most deleterious conditions—low speed and long track. The unaged laminate again exhibits a prolonged ultra-low regime of µ 0.10, but the transition to high friction now occurs after roughly 130 m, signaling exhaustion of any residual matrix smearing. Age-related acceleration is dramatic: the 7-day, 14-day, and 21-day samples climb to 0.43, 0.50, and 0.53, respectively, within the first few meters and hold those values, with the 21-day curve showing occasional spikes that point to intermittent fiber pull-out and unstable third-body layers. Overall, water-pressure aging systematically raises the steady-state friction level, shortens or eliminates the low-friction run-in, and makes the response less sensitive to sliding speed. The effect scales with immersion time because additional absorbed water (up to 10% mass gain) promotes matrix plasticization, hydrolysis, and interfacial debonding, all of which enlarge the true contact area and facilitate abrasive debris generation. Low sliding speed exacerbates this aging penalty by lengthening contact time and thermal exposure, whereas long tracks reveal the latent instability of any transient transfer films. Consequently, tribological performance of glass fiber composites in pressurized aquatic environments deteriorates rapidly beyond one week of immersion, with the 21-day material entering a consistently high-friction, wear-prone regime across all test conditions. An increase in COF was measured in hybrid polyester/glass/jute composites after wet aging, notably at reduced sliding velocities, according to Aranha et al. [26]. The study indicated that water aging prohibited the growth of transfer films, and specimens switched from low initial COF to high steady state COF values (from 0.12 to 0.50). The same was seen in this study where, for 50 mm/s, COF increased suddenly after removal of a lubricous resin-rich layer, notably for aged samples, due to direct counterface–glass interactions and interface weakening. For instance, Xu et al. [33] revealed that carbon fabric/polyetheretherketone (CFF/PEEK) composites demonstrated a reduction in tensile retention to approximately 85% after hygrothermal aging exposure at 70 °C, which was caused by water-induced matrix plasticization and interfacial weakening. In a related line of research, a study by Nan et al. [34] emphasized that aging in seawater caused physical and chemical matrix deterioration of an FRP composite, including resin swelling, interfacial detachment between matrix and fibers, and matrix hydrolysis, which, in total, contributed to deteriorated mechanical behavior. These results concur with increases in friction coefficients and wear rates for pressurized water-immersion-aged glass fiber-reinforced composites that have been observed.

**Figure 5:**
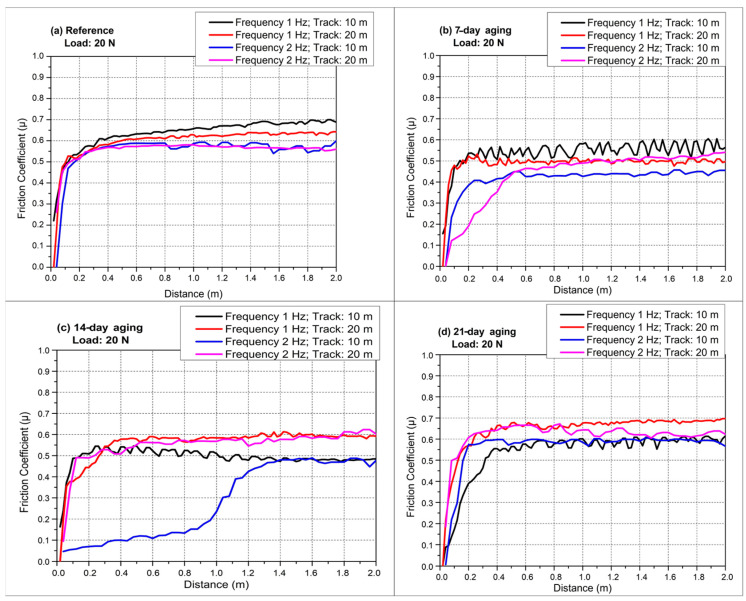
Comparison of COF values in ball-on-disc wear tests at 20 N load with various aging durations in first 2 m sliding distance: (**a**) reference, (**b**) 7-day aging, (**c**) 14-day aging, (**d**) 21-day aging.

Figure 5a, which represents the reference laminate without any aging, shows that the coefficient of friction (COF) rises erratically, especially at 100 mm s^−1^, until roughly the 20 m mark, before stabilizing, a behavior indicating that insufficient microstructural compatibility and chemical bonding at the fiber–matrix interface delay the formation of a protective tribo-film; at a lower speed of 50 mm s^−1^, the COF remains low yet unsteady, reflecting weak interfacial resistance and elastomer-dominated surface compliance that favor irregular wear, fiber pull-out, and micro-delamination.

Figure 5b, corresponding to the composite aged for seven days under 10 bar hydrostatic pressure, shows the COF changed to a steady value of about 0.40 within the first 3–5 m under every test condition, demonstrating that pressurized water fills micro-voids in the resin and redistributes residual stresses, thereby reinforcing the fiber–matrix interface; the resulting rapid mechanical accommodation allows homogeneous wear-film formation even at a low sliding velocity.

Figure 5c, depicting the specimen aged for 14 days at 10 bar, delivers the most favorable running-in response: irrespective of speed and track length, the COF reaches a stable 0.43–0.45 µ after only 3 m and maintains it, implying that controlled water diffusion has produced optimum matrix plasticization, maximum interfacial cohesion, closure of micro-cracks, and balanced internal stresses, all of which foster an exceptionally uniform tribological film and minimize sudden roughening.

Figure 5d shows the laminate aged for 21 days at 10 bar enters the running-in phase rapidly (<5 m) yet exhibits pronounced COF oscillations, suggesting that excessive water uptake has over-plasticized the matrix and partially weakened fiber–matrix bonds; the consequent enlargement of microscopic voids, local fiber fractures, and transient delamination generate friction fluctuations, so although running-in ends quickly, mechanical integrity and surface stability are inferior to those of the 14-day-aged specimen.

### 3.2. COF Analysis of Reciprocating Wear Tests

**Figure 6:**
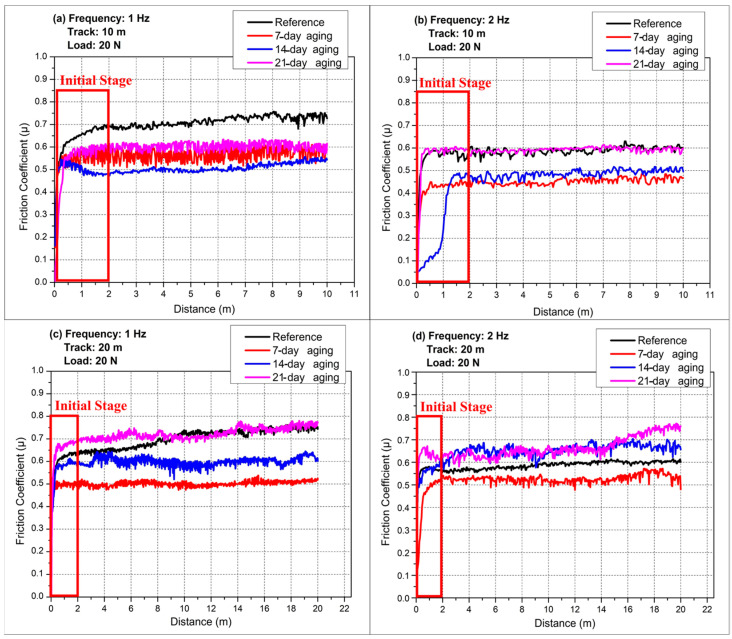
Effect of aging time on COF values at 20 N load in reciprocating wear tests: (**a**) 1 Hz; 10 m; (**b**) 2 Hz; 10 m; (**c**) 1 Hz; 20 m; (**d**) 2 Hz; 20 m. (The red rectangle shows the initial stage.)

Figure 6a captures the 1 Hz, 10 m reciprocating test, where every specimen reaches its steady-state friction in the first meter. In Figure 4, the plateau friction is reported as the mean ± standard deviation (μ ± SD) over the steady-state window after excluding the running-in segment (<10 m for ball-on-disc), whereas in Figure 5 the reciprocating friction is reported as the stroke-averaged μ (forward + reverse per stroke) computed over the steady-state window after the first 2 m, again expressed as mean ± SD. The unaged laminate quickly stabilizes close to 0.70, signifying a strong, debris-supported third body that survives the short stroke. Water-immersed panels display systematically lower plateaus: the 7-day coupon settles around 0.58, the 14-day one bottoms near 0.50, and the 21-day specimen climbs back to roughly 0.56. This reduction relative to the unaged reference is attributed to moderate water uptake that transiently plasticizes the polyester matrix and promotes a stable resin-rich transfer film, which lowers the real contact area and facilitates third-body accommodation, thereby depressing the apparent coefficient of friction under identical load and kinematics. Moderate aging therefore reduces the real contact area by plasticizing the matrix and promoting a smoother smearing of resin, yet extensive immersion gradually reverses this benefit as micro-cracks coalesce and fresh glass asperities are exposed. Figure 6b doubles the frequency to 2 Hz while retaining the 10 m stroke. A faster reversal rate trims the dwell time per half-cycle, weakening adhesion and narrowing the friction spread among conditions. The reference sample hovers just above 0.60, whereas 7- and 14-day-aged laminates range from 0.44 to 0.50, evidencing the same soft-matrix lubrication observed at 1 Hz. The 21-day curve, however, almost overlaps the virgin response, implying that severe hydrolysis now negates the velocity-induced adhesion drop by injecting brittle fiber fragments into the contact. Extending the track to 20 m at 1 Hz in Figure 6c accentuates fatigue of the tribo-layer. The reference composite displays a gentle upward drift from roughly 0.67 to 0.72 as accumulated debris thickens. The 7-day and 14-day specimens level off at lower values, around 0.60 and 0.55, but the 21-day coupon overshoots the virgin laminate, averaging nearly 0.75 and spiking intermittently when local delamination releases coarse fragments. The longer stroke exposes the fragile interface created by prolonged water uptake, allowing hard glass particles to dominate over any lubricious resin film. Accordingly, the monotonic rise in µ in ball-on-disc mode reflects a continuously swept annular contact that disperses and gradually thickens a third body as water-induced softening and debonding accumulate, whereas the confined, bidirectional reciprocating stroke exhibits a non-monotonic minimum at 14 days when a resilient resin-rich transfer film is most stable and debris is intermittently evacuated; by 21 days, over-plasticization and interfacial weakening inject brittle fiber fragments that are retained along the track, destabilize the film, and shift the dominant mechanism to abrasive third-body wear, lifting µ again. Figure 6d merges the most challenging conditions, 2 Hz and 20 m, and delivers the widest friction window. The unaged material stabilizes at 0.60, whereas 7-day and 14-day panels remain subdued near 0.50 and 0.55. In contrast, the 21-day specimen escalates to about 0.78 with pronounced oscillations, confirming that extensive aging converts the contact to an abrasive, fiber-rich regime whose severity outweighs the residence-time reduction offered by the higher frequency. We do not observe this oscillatory, high-friction response in the 7-day-aged laminate because its moisture uptake remains below the critical threshold for widespread interfacial debonding; moderate plasticization still supports a continuous resin transfer film and limits the generation/retention of brittle glass fragments, thereby suppressing third-body abrasion. Overall, reciprocating tests reveal a non-monotonic aging response. Initial immersion of up to 14 days lowers friction by plasticizing the matrix, smoothing the counterface, and favoring a thin resin transfer film. Beyond this threshold, continued water ingress, reflected in the mass gain from 72.2 g to 79.4 g, promotes interfacial debonding, crack coalescence, and fiber pull-out, driving friction above virgin levels, especially under long strokes where the brittle debris is repeatedly re-entrapped. Frequency alters the absolute coefficients by modulating contact time, yet the qualitative aging trends persist, underscoring that hydrothermal degradation is the primary governor of the tribological behavior of these glass–fiber laminates. Comparable trends have also emerged from the literature on how hydrothermal aging affects the frictional behavior of fiber-reinforced composites. Behera et al. [31] showed that, in SiC-filled short jute fiber/epoxy composites, the highest COF increments were realized through a combination of high frequency and large sliding distances subsequent to aging brought about by extensive matrix plasticization, interfacial delamination, and elevated fiber pull-out processes. The composites only retained 44% tensile strength after six months of soaking in alkaline solution at 60 °C, compared to 53% in distilled water, which emphasizes a significantly higher effect of alkalinity. This corresponds with Mula et al. [35], who reported that hydrothermal aging causes swelling stresses and interfacial delamination in GFRP composites, which is further accelerated by chemical reactions in an alkaline environment.

**Figure 7:**
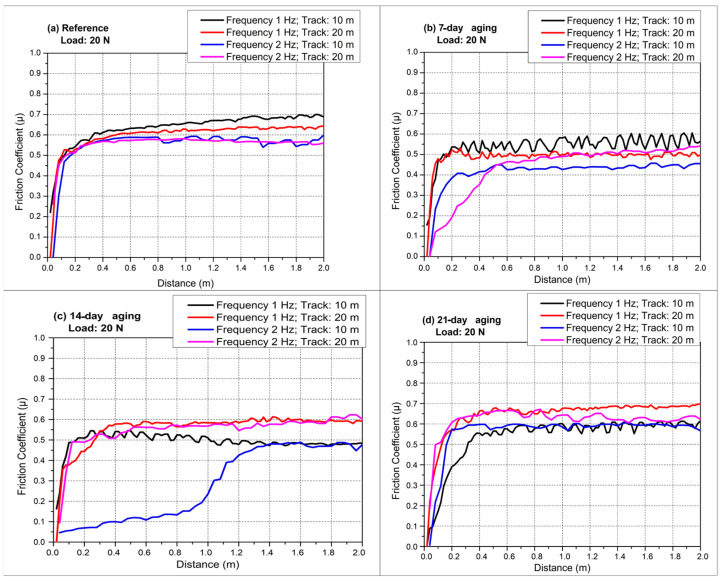
Comparison of COF values in reciprocating wear tests at 20 N load with various aging durations in first 2 m sliding distance: (**a**) reference, (**b**) 7-day aging, (**c**) 14-day aging, (**d**) 21-day aging.

Figure 7 compiles the reciprocating COF traces obtained under 20 N normal load while varying both stroke frequency (1 or 2 Hz) and total sliding distance (10 or 20 m), allowing the first two-meter running-in segment and the subsequent steady regime to be contrasted as a function of hydrostatic water-aging time. Figure 7 displays only the initial 0–2 m running-in interval; at longer sliding distances, the COF traces develop more pronounced debris-driven oscillations consistent with the later portions of the profiles shown in Figure 4, Figure 5 and Figure 6.

Figure 7a (reference laminate) reveals that all four tests display a steep, nearly vertical rise in COF within the first 0.15–0.25 m, signifying rapid seizure as dry glass asperities and fresh polyester are forced into contact; thereafter, the 1 Hz curves continue to climb more gradually until ≈2 m, reaching transient maxima of 0.72 (10 m track) and 0.68 (20 m track) before settling, whereas the 2 Hz curves peak lower, at ≈0.60, because the higher reversal rate shears nascent adhesive junctions before they mature. Beyond 2 m, a clear frequency hierarchy remains. 1 Hz > 2 Hz, while the longer 20 m stroke reduces each plateau by 0.03 owing to greater spatial dispersion of third-body particles and minor cooling between strokes. By contrast, the 14- and 21-day-aged coupons do not exhibit this stroke-length-induced drop because hydrolysis-driven interfacial debonding continuously injects brittle glass fragments into the contact; the longer travel re-entrains and retains this third body, so abrasive plowing and unstable tribo-films outweigh any dilution of debris, leaving the plateau μ unchanged or higher.

Figure 7b (seven-day-aged laminate) shows that the pressure-assisted infusion of water markedly softens the initial transition: although the COF still surges in the first 0.2 m, the overshoot is suppressed, and all curves approach their respective steady states by 1 m. The 1 Hz–10 m trace oscillates around 0.60–0.62 between 0.5 and 2 m, reflecting intermittent rupture of a developing resin transfer film; the 1 Hz–20 m curve follows a similar path but stabilizes 0.05 lower after the 2 m mark because the longer stroke dilutes debris concentration. At 2 Hz the short 10 m stroke levels offs at 0.50 within 0.8 m, while the 20 m counterpart declines slightly further to 0.46, indicating that high reversal frequency combined with extended travel distance most effectively mitigates adhesive junction growth once the interface has been modestly reinforced by seven-day aging.

Figure 7c (14-day-aged laminate) demonstrates an optimized interface whose running-in is virtually complete by 0.5 m for all but the 2 Hz–10 m test. In that outlier, the COF lingers near 0.35 until about 1.2 m before climbing towards 0.45, suggesting that the short stroke does not entrain sufficient debris to sustain a protective film until multiple cycles have roughened the contact. By contrast, the 2 Hz–20 m curve surpasses both 1 Hz traces beyond 0.8 m, plateauing near 0.60 because the combination of high cycle rate and long stroke continually injects fresh fragments that thicken an abrasive third body. The two 1 Hz curves, already at 0.50 by 0.3 m, diverge slightly after 2 m, the 10 m track settling at 0.55, the 20 m track at 0.52, underscoring the moderating influence of stroke length once micro-cracks have been sealed and matrix plasticity equilibrated by 14-day aging.

Figure 7d (21-day-aged laminate) exhibits the most aggressive early-stage behavior: all traces vault to µ > 0.50 within 0.1 m, but the 1 Hz–20 m and 2 Hz–20 m tests surge past 0.70 by 0.4 m, maintaining those high values with minor undulations throughout the first 2 m. These long-stroke runs continuously trap brittle fiber splinters liberated by over-plasticized resin, so third-body abrasion dominates almost immediately. In contrast, the 1 Hz–10 m and 2 Hz–10 m curves peak near 0.60 in the first meter, then hover around 0.55–0.58 beyond 2 m because the shorter path limits fragment retention even as the degraded interface still promotes adhesion.

Taken together, the first two meters of sliding capture the transition from asperity-controlled seizure to debris-mediated accommodation, and aging shifts both its rate and severity. Moderate water exposure (seven to fourteen days) attenuates the initial COF spike and shortens running-in by plasticizing the matrix and healing micro-voids, with high frequency plus long stroke giving the lowest steady values. Prolonged aging (21 days) oversaturates the resin, re-introducing a sharp rise and delivering the highest friction when long strokes perpetually recycle fiber fragments, showing that the interplay between water-induced interface degradation and reciprocating kinematics governs not only the steady-state COF but also the critical early-distance behavior.

### 3.3. Wear Damage Evaluation

**Figure 8:**
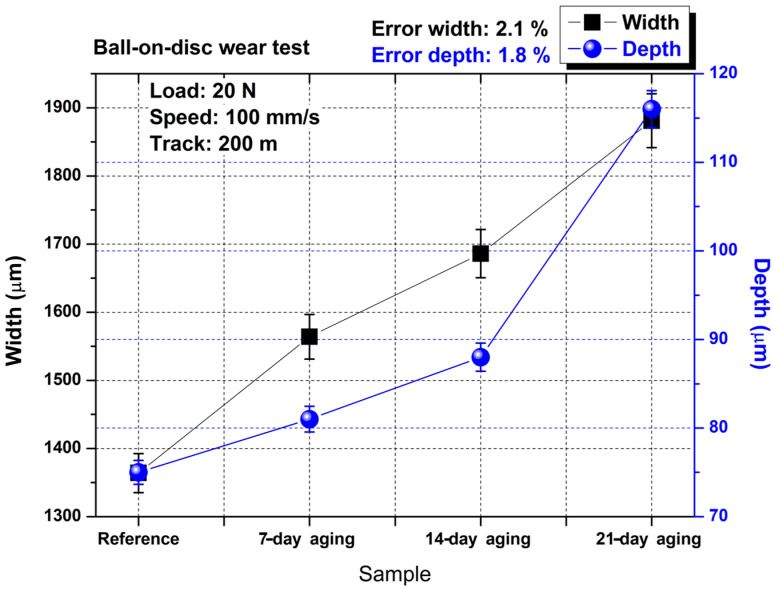
Comparison of width and depth values in ball-on-disc wear tests at 20 N load, 100 mm/s speed, 200 m track with various aging durations.

Figure 8 demonstrates that hydrostatic water aging progressively enlarges both the lateral (width) and vertical (depth) extent of the ball-on-disc wear scar: the mean width increased from 1.38 mm for the unaged laminate to 1.90 mm after 21 days, while the corresponding depth rose from 75 µm to 117 µm. Each data point represents the average of five independent profilometric cross-sections taken at evenly spaced locations along the track; the associated uncertainties, 2.1% for width and 1.8% for depth, were calculated from the dispersion of these replicate measurements and underscore the good repeatability of this method. The monotonic growth of both metrics mirrors the frictional trends in Figure 4c, where the steady-state coefficient of friction likewise climbs with immersion time, indicating that water-induced plasticization of the polyester matrix and debonding at the E-glass interface jointly promoted greater third-body entrainment, a larger real contact area, and deeper material removal. The sharp escalation between 14 and 21 days suggests that a critical moisture threshold was reached, beyond which interfacial cohesion deteriorated rapidly, accelerating abrasive and fatigue wear mechanisms and yielding the broadest, deepest grooves observed in the study.

**Figure 9:**
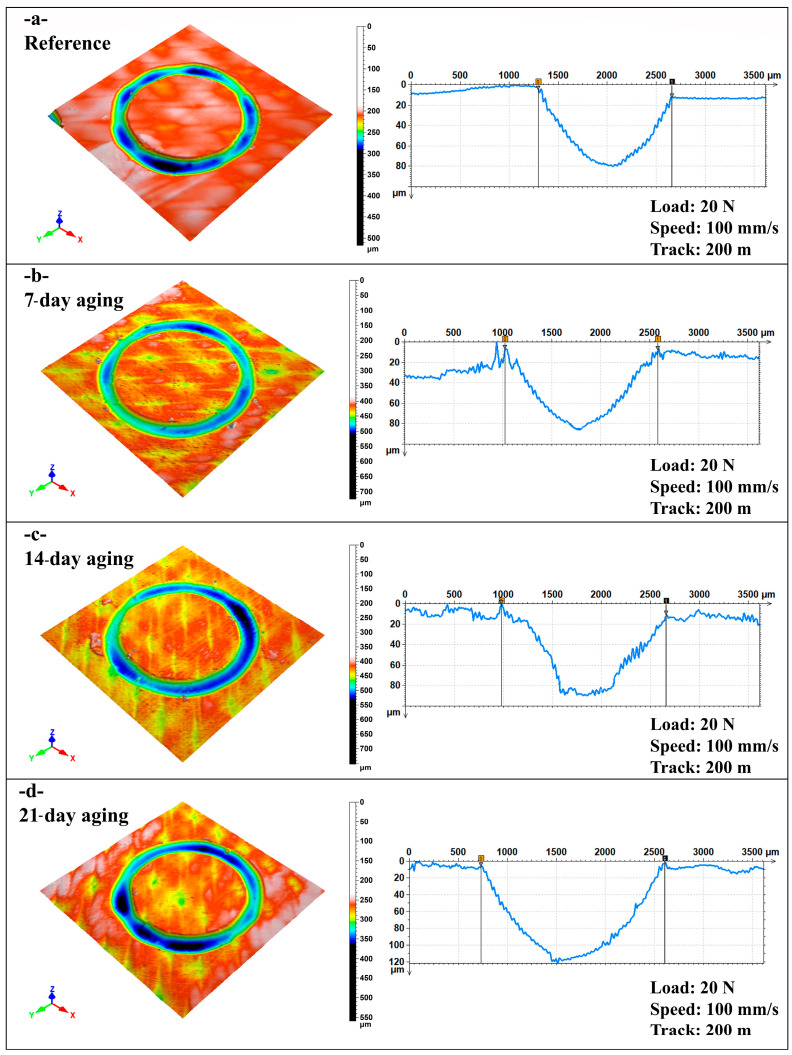
Comparison of profilometric 3D surface and cross-sectional images of ball-on-disc wear scars at 20 N load, 100 mm/s speed, 200 m track with (**a**) reference, (**b**) 7-day aging, (**c**) 14-day aging, (**d**) 21-day aging.

Figure 9a portrays the un-aged laminate after the 200 m ball-on-disc run and reveals a relatively narrow, shallow annular groove whose mean width (1.38 mm) and depth (75 µm) accord with the profilometric averages reported in Figure 7. The three-dimensional map shows a smooth U-shaped valley bordered by modest pile-up ridges, while the cross-section confirms a symmetric wear profile with gentle flanks; resin smearing largely blankets individual E-glass filaments, indicating that material removal was governed by mild micro-plowing and adhesive junction rupture rather than by aggressive fiber fracture.

Figure 9b corresponds to the specimen aged seven days at 10 bar and exhibits a visibly broader scar (1.55 mm) and a slightly deeper trough (85 µm). The surface relief now displays fine, directionally aligned plow marks inside the groove and a discontinuous lip of compacted debris at the outer edge, consistent with the higher steady-state COF seen in Figure 4c. A small protuberance in the section trace near the trailing edge indicates localized third-body accumulation, implying that moderate matrix plasticization has facilitated fiber–matrix debonding and the entrainment of larger wear fragments, without yet inducing wholesale fiber pull-out.

Figure 9c shows the laminate aged for 14 days reached the “critical” width (1.70 mm) identified in Figure 8. The wear valley was slightly deeper (90 µm) but its floor was flatter, suggesting that a stable tribo-film had formed and was periodically renewed as the track progresses. The 3D image reveals a smooth, continuous rim with minimal spallation, reflecting the optimum balance between matrix ductility and interfacial integrity; this morphology corroborates the highest frictional stability observed in Figure 4c, where the COF plateaus quickly and remains steady thereafter.

Figure 9d illustrates the 21-day-aged specimen and displays the most severe damage, with an average width of 1.90 mm and a pronounced depth approaching 115 µm. The cross-sectional profile is markedly V-shaped with steep walls and a deep central cavity, while the topography map shows ragged edges, fiber protrusions, and pronounced pile-up ridges produced by extensive fiber pull-out and brittle resin fracture. These geometric extremes align with the abrupt rise in COF and greater amplitude oscillations reported for this aging level, confirming that excessive water uptake weakens interfacial bonding, accelerates composite disintegration, and ultimately magnifies both lateral and vertical wear dimensions.

**Figure 10:**
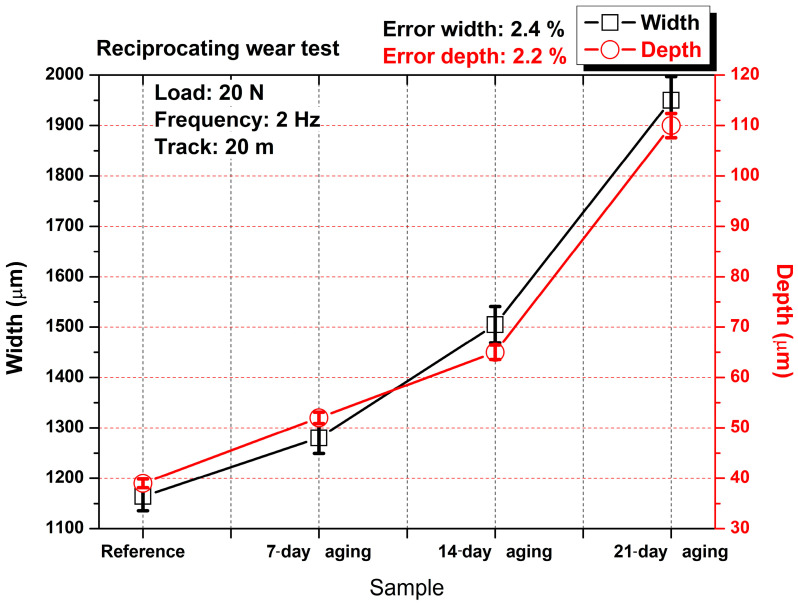
Comparison of width and depth values in reciprocating wear tests at 20 N load, 2 Hz frequency, 20 m track with various aging durations.

Figure 10 demonstrates that hydrostatic water aging progressively magnifies the reciprocating wear scar of the glass–fiber/polyester laminate; mean width grew from roughly 1.15 mm in the un-aged reference to about 1.95 mm after 21 days, while the corresponding depth rose from 40 µm to 110 µm. Each datum represents the average of five profilometric traces taken at evenly spaced positions along the track; the scatter of these replicates yields the quoted uncertainties of 2.4% for width and 2.2% for depth, confirming good measurement repeatability. The modest enlargement observed after seven days correlates with the slight drop in steady-state friction shown in Figure 5d, implying that limited matrix plasticization lubricated the contact yet left the softened surface more susceptible to lateral plowing. By 14 days, water uptake had reached a critical level that both reinstated higher friction (Figure 6d) and drove a 30% jump in scar dimensions, signaling the onset of interfacial debonding and abrasive third-body entrainment. Prolonged immersion for 21 days produced the steepest rise in both width and depth in parallel with the highest friction coefficient, evidencing severe hydrolytic degradation that weakened fiber–matrix cohesion, facilitated brittle fiber pull-out, and allowed the counterbody to excavate a broad, deep trough under the same mechanical loading conditions.

**Figure 11:**
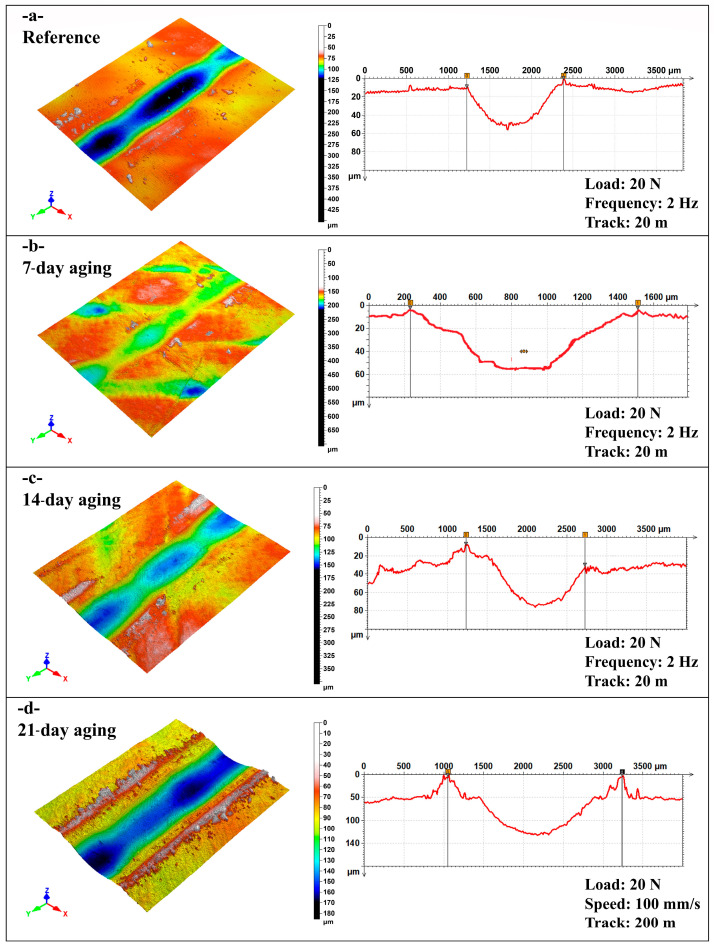
Comparison of profilometric 3D surface and cross-sectional images of reciprocating wear scars at 20 N load, 2 Hz frequency, 20 m track with (**a**) reference, (**b**) 7-day aging, (**c**) 14-day aging, (**d**) 21-day aging.

Figure 11a presents the 3D and cross-sectional topography of the unaged laminate following reciprocating wear testing. The wear scar appears relatively narrow and shallow, consistent with the width (1200 µm) and depth (40 µm) values in Figure 9. The cross-section reveals a well-defined, symmetric U-shaped groove, while the surface morphology remains largely smooth with minimal fiber exposure. This geometry suggests that the unaged composite maintained good fiber–matrix integrity, and that the wear mechanism was dominated by mild adhesive sliding and limited matrix smearing, with negligible fiber pull-out or interfacial failure.

Figure 11b corresponds to the laminate aged for seven days under 10 bar pressures and displays a moderately widened wear scar (1300 µm) with a slightly deeper valley (50 µm). The surface becomes more textured, with visible fiber traces and slight micro-spallation along the track. The cross-section shows an asymmetric trough, indicating onset of local interfacial weakening. This evolution suggests that moderate water absorption has started to reduce the matrix stiffness, promoting partial fiber–matrix debonding and increasing vulnerability to abrasive damage, in line with the modest rise in wear dimensions reported in Figure 9.

Figure 11c reflects the topography after 14 days of aging, where both wear width and depth show a pronounced increase (1500 µm and 70 µm, respectively). The 3D surface reveals a more rugged wear path with exposed fibers and noticeable material pile-up along the groove edges. The cross-sectional view is more irregular and asymmetric, indicating unstable third-body dynamics and progressive matrix degradation. These observations align with the COF increase and wear metrics in Figure 10, where hydrothermal exposure has sufficiently softened the matrix and degraded the interfacial strength, allowing enhanced fiber fracture and material loss under repeated reciprocation.

Figure 11d shows the surface morphology after 21 days of hydrostatic aging and demonstrates the most severe wear damage, with the widest (1950 µm) and deepest (115 µm) groove reported in Figure 10. The 3D surface map reveals significant fiber exposure, deep grooves, and discontinuous debris bands along the wear track. The cross-section shows an uneven V-shaped profile with steep flanks and deep penetration, indicating extensive fiber pull-out, matrix fragmentation, and unstable debris flow. These features confirm that excessive water absorption critically impairs interfacial bonding, resulting in dominant abrasive wear mechanisms and rapid deterioration of the composite surface under reciprocating sliding.

In agreement with results of the current study, Altaie et al. [36] emphasized the significance of wear mechanisms of adhesion and abrasion of composite materials subjected to reciprocating sliding testing. Their evaluation revealed that increased frictional interactions result in material transfer and debonding of filler particles in accordance with the increased wear track width and depth of aged composites subjected to reciprocating motion. Particularly, the dominance of the abrasive wear mechanisms with visible microcracks and debonding of filler particles reflects the progressive wear damage of prolonged aging scenarios of the current study. Correspondingly, Wierzbicka et al. [37] revealed that, in polyethylene-based composites, the coefficient of friction (COF) has a linear proportional increase with definite ratios of additive, and reciprocating wear tests revealed increased and deeper wear tracks as interfacial bonding deteriorated with cycling load. This result provides credence to the proposition that, when subjected to reciprocating motion, aged composite materials develop more widespread tribological wear manifestations due mainly to plasticization of the material and interfacial degradation, in accordance with the wear trends of the current study. Boron-strengthened epoxy composites were revealed by Apay and Kılınçel [38] to display lower friction coefficients of 0.2–0.4 with reference to pure epoxy of 0.4–0.6, which underlined the lubricating effect of nanopowders. In contrast, the current case reveals that prolonged aging disrupts such balances such that tribological deterioration via hydrolysis prevails and accentuates friction. This underscores an important absorption level of water beyond which tribological performance irreversibly deteriorates.

### 3.4. Wear Track SEM Damage Analysis

**Figure 12:**
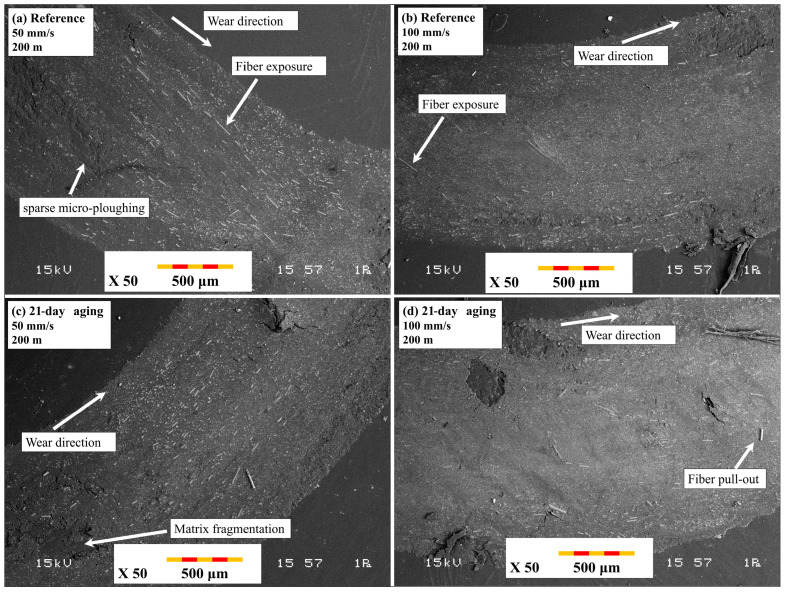
General wear track morphology after ball-on-disc tests (**a**) Reference, 50 mm/s, 200 m; (**b**) Reference, 100 mm/s, 200 m; (**c**) 21-day aging, 50 mm/s, 200 m; (**d**) 21-day aging, 100 mm/s, 200 m.

Figure 12 shows the overall wear track appearance for ball-on-disc tests. The images in Figure 12a,b are of the unaged reference specimens, and Figure 12c,d represents the wear tracks of specimens aged 21 days under 10 bar pressure. In Figure 12a,b, with test conditions of a 200 m sliding distance and 100 mm/s and 200 mm/s sliding speeds, reference samples have a generally smooth wear track with sparse micro-plowing marks and sparse fiber exposure, which signify an overriding adhesive wear behavior with an intact fiber–matrix interface. Conversely, in Figure 12c,d, where the aged samples were subjected to the same sliding distance but under 50 mm/s (c) and 100 mm/s (d) sliding speeds, extensive surface deterioration occurs. Particularly in Figure 12c, the decreased sliding speed increases interaction time per cycle, favoring extreme matrix fragmentation and prevailing fiber pull-out. The aged specimens have broader, deeper, and more irregular wear tracks due to the combined roles of matrix plasticization, interfacial de-adhesion, and intensified abrasive wear mechanisms, collectively further aggravated by extended water exposure and reduced sliding speeds. The deterioration occurred more extensively at 50 mm/s (Figure 12c), consistent with observations by Khakbaz et al. [29], who demonstrated that extended water exposure multiplies matrix plasticization and interfacial de-adhesion, leading to more aggressive abrasive wear under reduced-speed scenarios.

**Figure 13:**
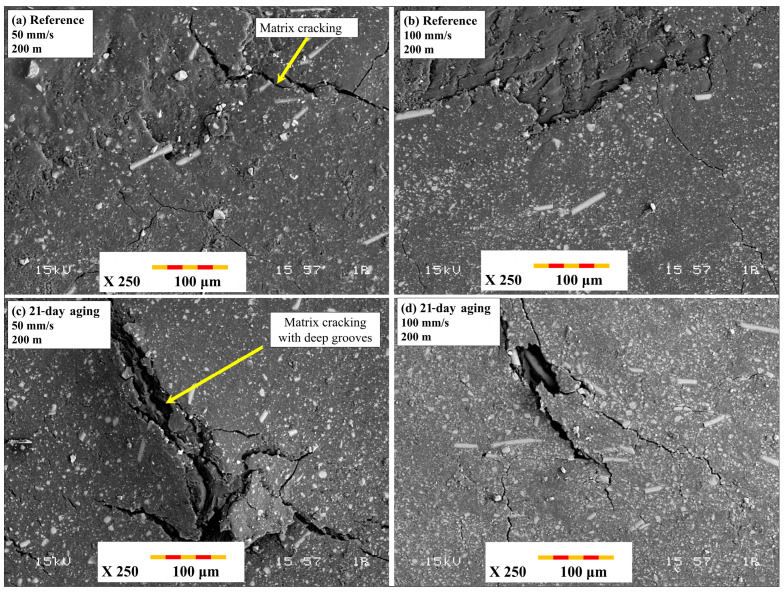
Detailed damage mechanisms after ball-on-disc tests: (**a**) reference, 50 mm/s, 200 m; (**b**) reference, 100 mm/s, 200 m; (**c**) 21-day aging, 50 mm/s, 200 m; (**d**) 21-day aging, 100 mm/s, 200 m.

Figure 13 reveals close-up SEM images of the dominant damage mechanisms of the ball-on-disc tests. Figure 13a,b represents the reference specimen tested at 50 mm/s and 100 mm/s, respectively, under 200 m of sliding distance. The figures illustrate smooth-surface characteristics of dominant adhesive wear and micro-plowing, with negligible fiber exposure and intact fiber–matrix bonding. In sharp contrast, Figure 13c,d represents the 21-day-aged specimens tested at 50 mm/s (c) and 100 mm/s (d). Severe third-body debris entrapment, matrix cracking, and fiber pull-out are noticeably evident. Particularly, in Figure 13c, which shows the combined effects of extended sliding distance and low speed, damage has proliferated with the appearance of brittle fractures and deep irregular grooves. This evolution reveals the extensive failure of interfacial adhesion due to previous aging, where lower sliding speeds further accelerate wear by extending the interfacial contact duration.

**Figure 14:**
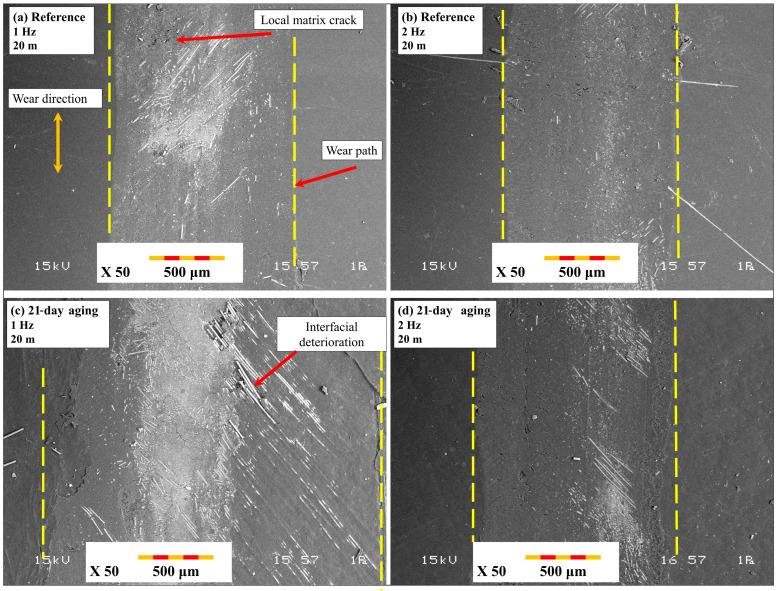
General wear track morphology after reciprocating tests: (**a**) reference, 1 Hz, 20 m; (**b**) reference, 2 Hz, 20 m; (**c**) 21-day aging, 1 Hz, 20 m; (**d**) 21-day aging, 2 Hz, 20 m.

Figure 14 demonstrates the typical morphology of wear tracks due to reciprocating wear testing. Figure 14a,b is indicative of reference specimens tested under 1 Hz and 2 Hz frequency and 20 m stroke length, respectively. Wear tracks are comparatively narrow with smooth appearances, indicating limited damage and optimum load distribution due to the retained interface. In sharp contrast, Figure 14c,d is characteristic of 21-day-aged specimens tested under 1 Hz (c) and 2 Hz (d) frequency and 20 m stroke length, respectively. Compared to the reference, aged specimens show astonishingly wide wear scars with prominent fiber exposure, disruption of fibers, and surface roughness. The low frequency in Figure 13c provides a larger duration of contact per stroke and hence more vigorous separation of the fibers and removal of materials. Aging and low-frequency reciprocating motion tend to significantly improve the degree of wear damage due to intensified interfacial deterioration.

**Figure 15:**
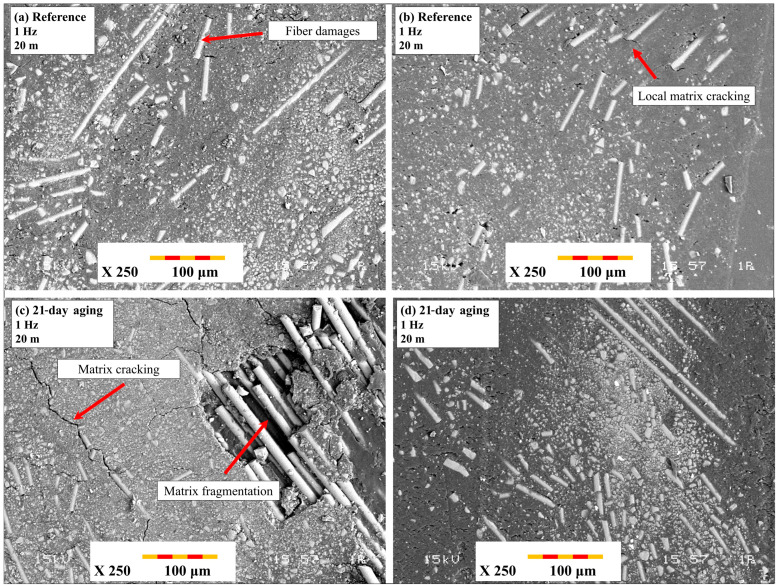
Detailed damage mechanisms after reciprocating tests: (**a**) reference, 1 Hz, 20 m; (**b**) reference, 1 Hz, 20 m; (**c**) 21-day aging, 1 Hz, 20 m; (**d**) 21-day aging, 1 Hz, 20 m.

Figure 15 reveals close SEM images of damage mechanisms of specimens tested under reciprocating wear tests. Figure 15a,b is indicative of the reference specimen surfaces under 1 Hz and 2 Hz frequency and 20 m track length. Wear surfaces indicate predominantly mild micro-plowing and minimal exposure of fibers, indicating good matrix integrity and negligible adhesive wear. On the other hand, Figure 15c,d is indicative of 21-day-aged specimens tested under 1 Hz (c) and 2 Hz (d) frequencies and 20 m sliding stroke. In these instances, extensive fiber pull-out, extensive matrix fragmentation, and deep grooves of an abrasive nature were recorded, more predominantly under 1 Hz frequency, as shown in Figure 14c. Long-term contact duration at lower frequency enabled premature interfacial failure to take place and combined the effects of hydrothermal degradation and reciprocating stress to induce an unstable third-body wear regime. These findings indicate the importance of water-driven interface deterioration and accentuation under reciprocating motion, and that they occur predominantly under lower frequency and extended stroke settings.

### 3.5. Factorial Contribution Analysis

The experimental design followed a full factorial scheme with three independent factors: (i) storage duration (days: 0, 7, 14, 21), (ii) sliding frequency (1 Hz and 2 Hz), and (iii) track length (10 m and 20 m). This resulted in 16 test conditions. Each condition was measured once (*n* = 1), and therefore no replications are available within factor-level combinations. Descriptive statistics complemented with bootstrap confidence intervals (95% CI, α = 0.05) were employed to provide an estimate of variability. Standard assumption checks for normality and homogeneity of variance could not be conducted due to the absence of replication. Effect sizes were expressed in terms of the relative contribution of factors to the observed variation in mean COF, while no post hoc comparisons were applicable under the current design. Because the fixed-factor full factorial design is saturated (Error DF = 0), the analyses reported here are descriptive variance decompositions (SSᵢ/SS_total, %); no *p*-values were computed, and we therefore avoid ‘statistically significant’ claims, restricting interpretation to effect contributions and trends.

Table 1 in this study was created in order to investigate the effects of the days, ball speed (mm/s), and ball track (m) parameters and their interactions on the coefficient of friction (COF) obtained from the surface of the laminated composite material as a result of the ball-on-disc test. According to the results in the table, the total explanatory power of the model is 100% and all variation is captured by this factorial model. 60.53% of the total variance belonged to the main factors (days, ball speed, and ball track). Here, the dominant effect came from the ball track variable (28.70%). This shows that the COF value was largely related to the contact distance. Ball speed also contributed 24.64%, revealing that the speed of the sliding surfaces was a determining factor in the tribological response. Although the days parameter had a lower contribution rate of 7.19%, this does not mean that the effect of material aging was limited because the effect of this factor increased in interactions.

Binary interactions accounted for 36.17% of the total variance. The most striking interaction was days × ball speed, contributing 31.66%, which strikingly demonstrates how aging time and speed alone affected COF. This confirms that samples aged at low speeds were characterized by high friction, meaning that interface deterioration was rapidly activated. Other interactions (e.g., days × ball track and ball speed × ball track) provided more limited contributions (3.88% and 0.62%). Regarding the triple interaction, the combination days × ball speed × ball track contributed 3.31%.

**Figure 16:**
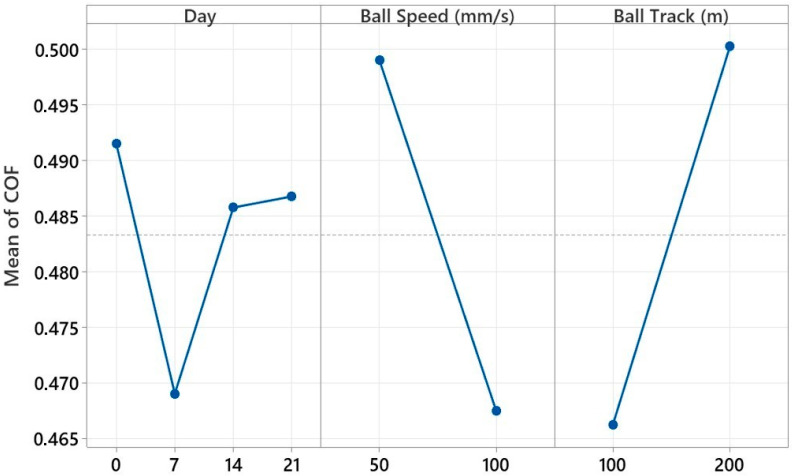
Mean effect plots of COF for ball-on-disc test.

The average effect graphs given in Figure 16 show the changes in the coefficient of friction (COF) in the ball-on-disc test depending on the parameters of day number, ball speed, and track length. In the day number parameter, the COF, which was approximately 0.490 on day 0, decreased on day 7 and reached a minimum value (0.469). On days 14 and 21, it showed an increase again, reaching close values (0.485–0.487). While the highest COF (0.498) was observed at a ball speed of 50 mm/s, the COF decreased as the speed increased to 100 mm/s, reaching a minimum level (0.467). In the track length parameter, the lowest COF (0.466) was obtained at 100 m, and the maximum value (0.501) was recorded at 200 m. These results reveal that the coefficient of friction was sensitive to all three parameters examined, and especially low speed and long track length can cause an increase in COF by changing the wear mechanisms on the surface.

Table 2 shows the sources of variance in track width measured in ball-on-disc tests and the contribution of each factor to the total variance. The model explains 100% of the total variance, demonstrating that the factors of days, ball speed, and ball track, as well as their interactions, fully model the changes in track width. Among the main effects, the factor “ball track” had the highest contribution rate, at 62.39%. This numerically confirms that increasing friction distance caused an increase in track width (Seq SS = 499.496). The second most influential main factor, “days,” contributed 21.31%, suggesting that increasing aging time had an effect on track width (Seq SS = 170.606). The factor “ball speed” had a lower effect, with a contribution of 2.47% (Seq SS = 19.811).

Binary interactions accounted for 11.51% in total. Among these, days × ball track showed the highest binary interaction value at 5.68% (Seq SS = 45.460), proving that the combination of aging time and friction distance was effective in increasing wake width. Ball speed × ball track came in second at 3.51% (Seq SS = 28.140), and this combination was observed to cause moderate increases in wake width. The days × ball speed interaction contributed less to the wake width, at 2.32% (Seq SS = 18.565).

The triple interaction (days × ball speed × ball track) showed the lowest effect, with a contribution of 2.32% (Seq SS = 18.536), confirming that the variance in the system is largely explained by the main effects and pairwise interactions.

**Figure 17:**
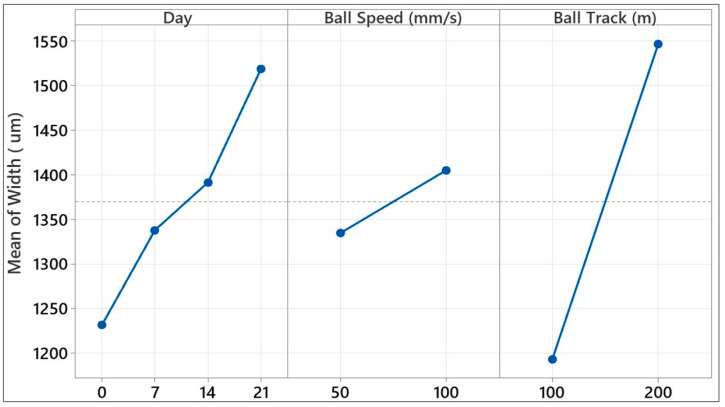
Main-effect plots for track width in ball-on-disc tests showing factor levels of aging time (0, 7, 14, 21 days), ball speed (mm/s), and sliding distance (m).

Figure 17 visually presents the effects of three key input parameters, aging time (days), ball speed (mm/s), and sliding distance (m), on track width obtained in ball-on-disc wear tests. Such average effect plots demonstrate the independent effect of each factor level on the response variable and support the interpretation of the contribution rates obtained from the analysis of variance. The plot shows a clear trend toward increased track width as aging time increased. Consequently, more material was removed from the contact surface, which was directly reflected in the track width. The ball speed effect curve had a more level profile and had a comparatively small range of variation. It tended to have narrower track widths at lower speeds (50 mm/s), but the track width also decreased as the speed increased to 100 mm/s. This means shorter contact times at lower speeds made weaknesses around the interface of the fibers more apparent and hence more damage appeared on the surface. Shorter contact times at greater velocities may limit damage spread across the surface, thus narrowing scarring. Wear scar width increased hugely as the sliding distance increased. This positively sloped linear relationship means more volumetric damage happened when the material underwent more prolonged mechanical stress and hence this damage happened further across the surface, more so around weakened regions of the matrix–fiber interface. The ball track variable had the highest contribution (Table 2), and this graph categorically proves this finding.

Table 3 presents the sources of variation in track depth measured in the ball-on-disc tribological experiment and the percentage of each factor contributing to the total variance. The model accounts for 100% of the total variance, confirming that the parameters days, ball speed, and ball track, and their interactions, account for all track depth variability. From the main effects, we have the dominant variable as the ball track factor, with a percentage contribution of 42.16%, showing that an increase in sliding distance resulted in deeper wearing on the material surface (Seq SS = 2782.56).

The second-most-contributing main factor was days of aging, with a percentage contribution of 32.16% (Seq SS = 2122.69), showing an increased rate of depth loss with an increase in aging time. Ball speed had a very small percentage contribution of 0.27% (Seq SS = 18.06), showing an attenuated effect of ball speed change alone on track depth. The combined effect of the binary interactions was computed as 23.56%.

The most prominent contribution was the days × ball speed combination, 13.14% (Seq SS = 867.19), which shows that the interaction effect of aging time on loss of depth under varying speed conditions outweighed the single-factor effects. The days × ball track interaction, contributing 5.37% (Seq SS = 354.69), reaffirms that increases in depth were greater at longer sliding distances and greater sample ages. The ball speed × ball track interaction, contributing 5.05% (Seq SS = 333.06), shows that the speed parameter affected deep wear at long sliding distances. The triple interaction (days × ball speed × ball track) accounted for 1.85% (Seq SS = 122.19). This small contribution indicates that track depth change was largely accounted for by the main effects and binary interactions, but triple interactions may be important in some particular combinations.

**Figure 18:**
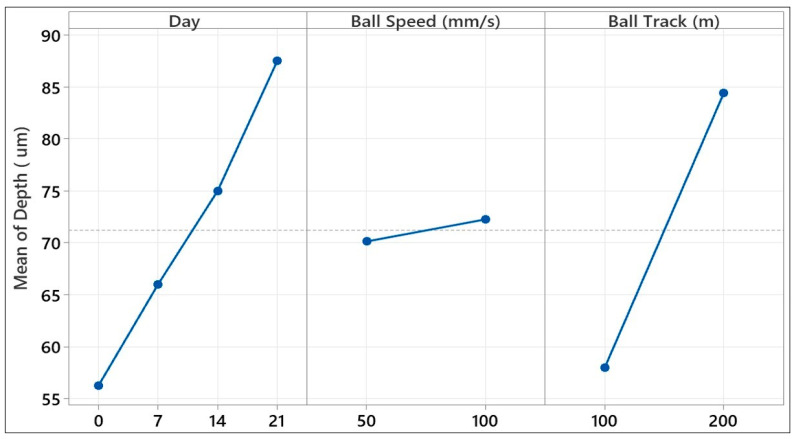
Main-effect plots for track depth in ball-on-disc tests across aging time (0, 7, 14, 21 days), ball speed (mm/s), and sliding distance (m).

Figure 18 visually demonstrates the individual effects of the three key test parameters on the wear scar depth of a glass fiber-reinforced composite material. The graph shows that the aging time and sliding distance variables, in particular, had dominant effects on scar depth. An increase in scar depth was observed as the aging time increased, particularly in samples exposed to hydrostatic water for 14 and 21 days. Similarly, an increase in sliding distance caused a linear increase in scar depth, indicating that tribological stresses penetrated deeper layers as the friction time increased. In contrast, the effect of ball speed was quite limited, and the change in scar depth at different speed levels was negligible. These graphical observations are consistent with the contribution rates obtained from the factorial analysis of variance; it is understood that sliding distance and aging time contribute to the explanatory power of the model at 42.16% and 32.16%, respectively. In summary, these results provide evidence of environmental aging time and mechanical strain as the governing parameters of the deep surface degradation of glass fiber-reinforced composites and confirm that they need to be accurately controlled, particularly under severe service conditions like submarine or underwater applications.

Table 4 presents the results of a factorial analysis of variance determining the effects of the main input parameters and their interactions on the coefficient of friction (COF) measured during reciprocating tribological testing of a glass fiber-reinforced composite. This analysis determines governing factors leading to tribological behavior through statistical evaluation of the effect of each variable and their combinations on COF. The total explained variance is 100%, which means all variability in the COF was successfully accounted for by the model. This further implies that all that is required to describe tribological performance is a collection of three parameters that were accounted for in this experimental design and their interaction, without any additional input variables. The main effects account for 33.98% of the total, which indicates that interactions of two, or the interaction of all three, of these variables are important contributors.

Aging time had the single largest influence on COF (21.21%). This verifies that aging has a deleterious effect on tribological performance. Indirectly, test frequency (7.20%) also dominated COF through changing contact times and loading application rates. At low frequencies, COF increased due to increased contact time and accumulation of thermomechanical stress, but at high frequencies, COF reduced due to an inhibition of adhesion effects on the surface. Sliding distance accounted for the total amount of 5.57% of the damage that occurred during the test.

Binary interactions accounted for 47.04% overall, illustrating how an interaction between the parameters influenced the outcomes. The relatively high contribution level of Days × Frequency (20.36%) reveals that frequency’s effect increased in aged samples. This synergistic effect reveals the sensitivity of tribological systems to dynamic circumstances. Taking aging time and sliding distance into account, the contribution level of Days × Track (20.03%) produced an important ascending trend on the COF. Frequency × Track shows that surface wear during high-frequency, long-distance testing influences COF. Frequent contact at high frequencies resulted in unstable third-body debris accumulation on the surface over long distances, which created more friction.

The triple interaction (18.98% contribution) was the only triple interaction within the model and had a considerable contribution. This shows that, during high-frequency and long-distance conditions, materials aged for a longer time demonstrate frictional behaviors which become highly unstable and complex. In sum, this analysis identifies that the COF in reciprocal tribological testing highly depends not only on individual but also on binary interactions, especially aging time and frequency, and aging time and sliding distance. In addition, the effect from the triple interaction was also considerable. These results show that the effects of environmental aging on the tribological performance of glass fiber-based composites is highly determined not only by effects of individual test parameters but also by their interactions. As a result, considerable attention needs to be paid to these multiple interactions for tribological life estimations and designing materials.

**Figure 19:**
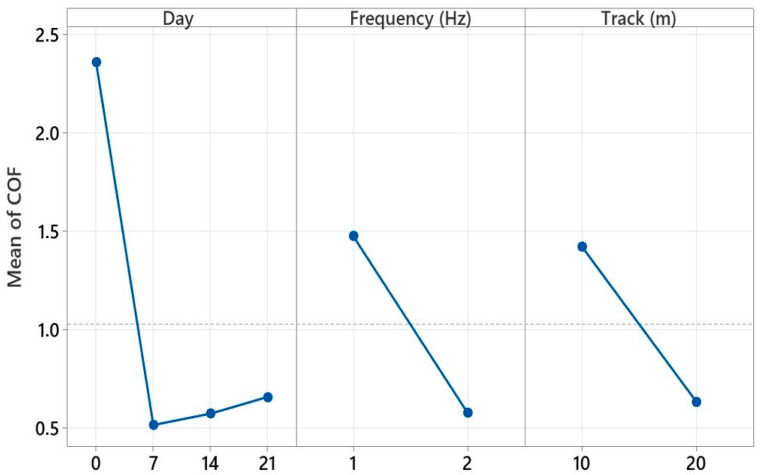
Mainn-effect plots of COF for reciprocating tests.

Figure 19 visually presents the average effects of three key experimental parameters on the coefficient of friction (COF) obtained during the reciprocating wear test of a glass fiber-reinforced composite material. The graph aims to identify the effective variables for tribological performance by evaluating the individual effect of each parameter on the COF in isolation. It shows that the COF value increased as the aging time increased. An increase in COF was observed, especially after 14 and 21 days of hydrostatic aging. In aged samples, the load-carrying capacity decreased due to the surface becoming more brittle, and the contact area became more irregular, leading to an increase in the COF. The curve for the frequency parameter shows a smoother increase. In low-frequency (1 Hz) tests, because the contact time was longer, the effects of adhesion and deformation on the surface became more pronounced, and the COF values remained high. On the other hand, as the frequency increased (2 Hz), the microstructural adhesion effects weakened as the contact time decreased, and the coefficient of friction remained lower. However, the effect of frequency may vary with the effect of aging; this explains the combinations that stood out, particularly in advanced interaction analyses. The average influence curve for the sliding distance (Track) parameter similarly showed that the COF increased with increasing distance. This can be explained by the increased cumulative damage to the material surface with increasing friction and time, the reintegration of third-body particles into the surface, and the decrease in stability at the interface. Overall, Figure 18 clearly demonstrates that aging time was the single most influential factor on the COF, while parameters such as frequency and sliding distance offered more limited but complementary effects. The graph also demonstrates high agreement with the contribution rates determined in the factorial analysis and visually confirms the complex response of the tribological system to environmental aging and mechanical stress. These findings demonstrate that tribological life predictions under reciprocating test conditions should be evaluated not only with individual variables but also with consideration to the discontinuous and multifaceted effects of these variables.

Table 5 provides a factorial analysis of variance (ANOVA) which investigates how the wear track width values from reciprocating tribology tests varied under three fundamental test parameters and interactions between the parameters. This analysis determines the contribution percentages of the factors affecting the trace width, thereby quantitatively revealing which parameters played a dominant role in terms of the spread of surface wear. The main effects contributed a total of 47.93%, with the sliding distance showing the highest contribution at 26.93%. This finding indicates that the width of the wear track developed directly in relation to the test duration and the number of contact repetitions. The aging time ranked second with a contribution rate of 13.26%. Although the contribution of the frequency parameter was more limited at 7.74%, it reveals that the speed of the loading cycles can affect the track width. A long contact time at low frequency can increase the width by causing more friction and material transport on the surface, while this effect may be limited at high frequency due to shortened contact time. Interactions contributed a total of 38.49%, indicating the importance of multiple-factor effects on wear track width. In particular, the Days × Frequency (18.32%) and Days × Track (14.30%) interactions stand out. This indicates that aged materials were more sensitive to frequency and sliding distance, and that track width varied depending on test conditions due to interfacial weaknesses. The interaction between frequency and track, on the other hand, contributed a more limited effect of 5.87%. The triple interaction, Days × Frequency × Track, was one of the notable contributors in the model, contributing 13.58%. This indicates that the material’s tribological response was not only dependent on independent variables but also on parameter combinations to a prominent degree. Especially as the aging time increased, it is understood that different frequency and distance values led to more complex and unpredictable effects on the track width. In conclusion, this factorial analysis shows that the wear track width during reciprocating tests was most affected by sliding distance and aging time, but these effects are complex due to frequency and, especially, multi-parameter interactions. Structural weaknesses that develop in composite material systems under environmental aging conditions, when combined with dynamic test conditions, make it difficult to predict wear behavior; this necessitates multi-parameter evaluations during the design phase.

**Figure 20:**
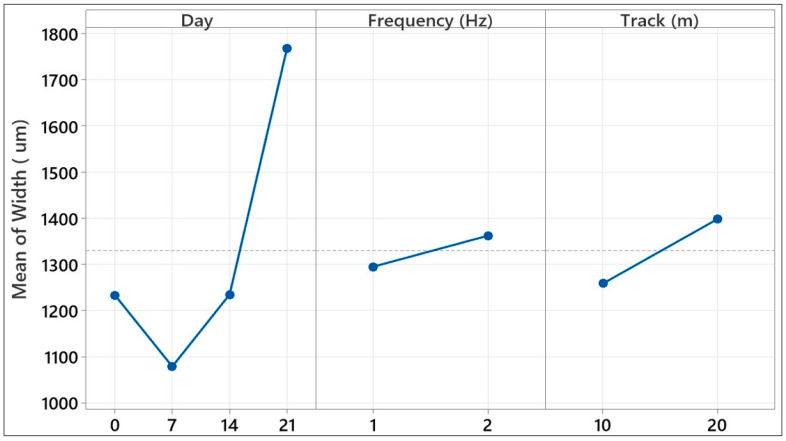
Main-effect plots of track width for reciprocating test.

Figure 20 graphically illustrates how wear track width values from reciprocal tribological testing of glass fiber-reinforced composite materials were altered with three fundamental test parameters. From this graph, individual assessment of each parameter’s average effect on track width is possible to see, as well as an indication of surface wear behavior. The variable aging time in the graph explicitly manifests an increase in wear track width with longer aging times. Specifically, track width considerably increased in samples aged for 14 days and 21 days in pressurized water. Considering the frequency parameter, it can be noticed that trace width was elevated during low frequencies (1 Hz) and declined with an increase in frequency. This pattern indicates that, under low frequencies, a higher contact time generates increased traces with surface wear, but under high frequencies, a restriction effect occurs due to decreased contact time. In this scenario, frequency becomes a key, albeit secondary, variable that actively forms wear progression. The sliding distance variable, however, presents as the key parameter determining track width. As sliding distance increased, a linear and consistent growth of track width was seen. For the prolonged duration of this test, more cumulative deformation, microstructural crack formations, and contaminant transfer on the surface occurred, causing an increase in track width. In general, Figure 20 portrays that aging time and sliding distance present overriding influences on track width, whereas frequency creates more constrained but major changes than those two variables. In this scenario, simultaneous assessment of environmental aging and mechanical loading conditions is important in correctly estimating the surface durability and wear behavior of glass fiber-reinforced composites.

Table 6 reveals the importance of the three principal test parameters and their two-way and three-way interactions on wear track depth values from reciprocal tribological experiments of glass fiber-reinforced composite materials through a factorial analysis of variance (ANOVA). These principal effects account for a total of 50.97% towards wear track depth, which decisively determines it. Of all these impacts, aging time contributed the largest proportion, 27.38%, which implies that environmental aging (primarily water exposure under pressure) caused deterioration of the fiber–matrix interfacial region within this composite structure, rendering it more prone to further wear. Sliding distance ranked second with a contribution of 18.92%, indicating that cumulative micro-damage on the surface increased track depth as contact time increased. The frequency variable contributed only 4.67%, indicating that its effect on track depth was indirect and secondary. The two-way interactions contributed a total of 35.66%, with the Days × Frequency (14.49%) and Days × Track (13.91%) interactions standing out. These results indicate that aged materials were more sensitive and brittle under dynamic loading conditions (frequency) and test duration (distance). The Frequency × Track interaction played a more limited role with a contribution rate of 7.26%, but it could still be effective in increasing surface fatigue. The triple interaction Days × Frequency × Track showed an effect with a contribution rate of 13.37%. This situation reveals that aged materials exhibit more unstable and unpredictable wear behaviors under harsh conditions such as high frequency and long sliding distance. In particular, it was observed that wear depth increased when these three parameters changed simultaneously. In conclusion, this factorial analysis demonstrates that wear track depth under reciprocating test conditions is determined not only by individual parameters but also by the complex interactions between these parameters. While aging time and sliding distance have the most dominant effects, the depth-increasing effect of frequency was more pronounced when evaluated in conjunction with aged structures.

**Figure 21:**
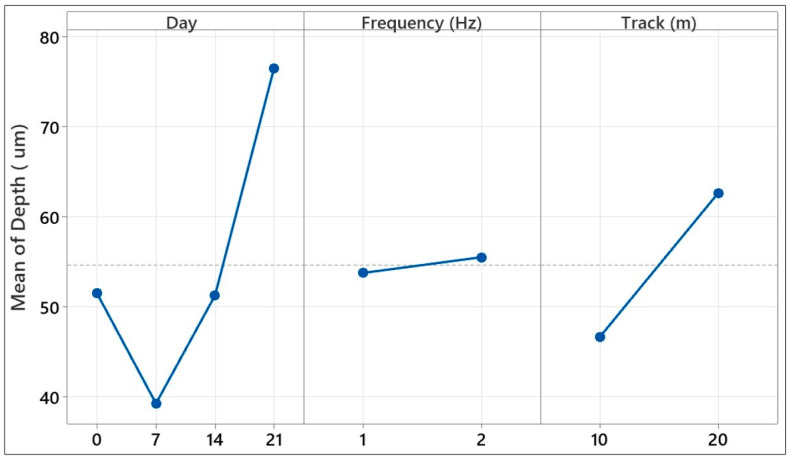
Main-effect plots of track depth for reciprocating test.

Figure 21 shows the average effects of three basic experimental parameters on the wear track depth formed during reciprocating tribological tests on glass fiber-reinforced composite materials. The aging time (Days) variable in the graph shows the dominant increase trend on the wear depth. In particular, an increase in the groove depth was observed under 14- and 21-day aging conditions. As the aging time increased, these deteriorations became more permanent and widespread, leading to an increase in subsurface deformations. The parameter of frequency had a less wide but noticeable influence on trace depth. In low frequency values (e.g., 1 Hz), the application time of loads was greater, leading to greater accumulation of thermomechanical stress on the surface, which led to deeper wear traces. As the frequency increased, the contact time decreased, allowing the damage to remain more superficial. However, it can also be understood from other analyses that the effect of frequency may increase when evaluated in conjunction with aging. The sliding distance also had an effect on wear depth. As the sliding distance increased, a linear increase in groove depth was observed. An extended test duration led to more cumulative micro-wear, fiber breakage, and matrix deformation on the surface, which accelerated the development of deep damage on the material surface. Overall, Figure 21 shows that aging time and sliding distance have a dominant effect on groove depth under reciprocating test conditions, while the frequency variable provides a secondary but complementary contribution to these effects.

In this study, the friction coefficient (COF), wear track width, and depth parameters obtained from ball-on-disc and reciprocating tribological tests conducted after exposing glass fiber-reinforced polymer composite materials to water at a pressure of 10 bar for different durations (0, 7, 14, and 21 days) were evaluated using factorial analyses of variance (ANOVA). In the ball-on-disc test, the sample surface was examined under continuously changing circular contact. In this test, the fact that contact was established with a different region of the surface at all times led to the effects of local damage caused by water absorption being distributed, resulting in a more limited average surface response. According to the ANOVA analyses, the dominant parameter affecting the COF was the ball track, while the effect of aging time remained at a lower level. Similarly, in terms of track width and depth, the sliding distance emerged as a factor that increased cumulative damage on the surface. The ball-on-disc test’s wider contact area and lower local stress intensity appear to have partially masked the effects of aging-induced weakening on wear behavior. In contrast, in the reciprocating test, the load was applied repeatedly to the same area, allowing the accumulation of local damage to be observed more clearly. In particular, the aging time had a high contribution rate in all tribological parameters (COF, track width, and track depth). In the ANOVA analyses, aging time emerged as the most dominant or second-most influential factor in terms of both the COF and wear geometries, while its interactions with parameters such as frequency and sliding distance also reached high contribution rates. Reciprocating testing demonstrated superior sensitivity in revealing the material’s response to environmental degradation. As a result of all evaluations, it was clearly revealed that the tribological behavior of glass fiber-reinforced polymer composites exposed to water for specific periods of time under 10 bar pressure was directly related not only to external loading conditions but also to the contact characteristics of the applied test method. These effects were also numerically supported in ANOVA analyses; aging time was one of the variables that contributed the most to most tribological parameters, both as a main effect and in interactions with frequency and distance. In contrast, in the ball-on-disc test, contact occurred over broader and continuously changing surface areas, causing local weaknesses to spread out over the test period and result in an average effect. This situation caused the effects of aging reflected on the surface to be “diluted” throughout the test, leading to less variability in the tribological response, particularly in terms of the COF, compared to the reciprocating test. Therefore, to reliably evaluate the tribological behavior of glass fiber-reinforced composites under water-related environmental aging conditions, it is necessary to consider not only the type of surface loading but also dynamic parameters such as whether the contact mechanism is local or distributed, the intensity of the stress field, the number of contact cycles, frequency, and sliding distance. Especially in environmentally demanding applications such as submarine structures, underwater pipelines, and tribomechanical systems operating in wet environments, it is recommended to use high-sensitivity methods such as reciprocating tests to accurately predict the service life of the material. In this context, the study strongly emphasizes the necessity of selecting test systems that are suitable not only for mechanical test parameters but also for the types of microstructural weakening that interact with the environmental life of the material.

## 4. Conclusions

Pressurized water immersion at 10 bars progressively degrades the tribological performance of cross-ply E-glass/polyester laminates and establishes a clear aging threshold. After 21 days, the laminates gained 10% mass (72.2 g to 79.4 g), coincident with higher steady-state friction in ball-on-disc (BOD) tests and a shortened/vanished low-µ run-in; at 100 mm s^−1^ and 200 m, the reference plate COF leveled off near 0.40 and 0.47, whereas the COF of aged coupons clustered around 0.49 to 0.52 and showed earlier stabilization. Wear geometry corroborated this trend; BOD scar width and depth increased monotonically from 1.38 mm to 1.90 mm and 75 to 117 µm between 0 and 21 days. Reciprocating tests revealed a non-monotonic response; moderate aging (14 days) minimized µ to 0.50, but prolonged exposure produced the harshest regime, with µ climbing to 0.78 under 2 Hz/20 m and pronounced oscillations. Factorial ANOVA isolated mechanisms; in BOD, track depth variance was dominated by sliding distance (42.16%) and aging (32.16%), with a strong Days × Speed interaction (13.14%); reciprocating COF was governed by aging as the largest main effect (21.21%), amplified by Days × Frequency (20.36%) and Days × Track (20.03%) interactions. The study’s novelty lies in pairing controlled hydrostatic aging (10 bar) with dual sliding kinematics under identical load to reveal time-dependent transitions in third-body abrasion and interfacial debonding. Future work should couple micro-CT/SEM of subsurface damage with DMA/nanoindentation to quantify plasticization; model moisture–diffusion/tribology coupling for life prediction; probe combined pressure–temperature–salinity environments; and evaluate interface-stabilizing strategies (sizing, nano-fillers, hydrophobic coatings, etc.) to delay the ≥14–21-day deterioration window.

## Figures and Tables

**Table 1:** Factorial contribution analysis of COF from ball-on-disc test.

Source	DF	Seq SS	Contribution	Adj SS	Adj MS
Model	15	0.016111	100%	0.016111	0.001074
Linear	5	0.009752	60.53%	0.009752	0.00195
Days	3	0.001159	7.19%	0.001159	0.000386
Ball Speed (mm/s)	1	0.003969	24.64%	0.003969	0.003969
Ball Track (m)	1	0.004624	28.70%	0.004624	0.004624
Two-Way Interactions	7	0.005827	36.17%	0.005827	0.000832
Days × Ball Speed (mm/s)	3	0.005102	31.66%	0.005102	0.001701
Days × Ball Track (m)	3	0.000625	3.88%	0.000625	0.000208
Ball Speed (mm/s) × Ball Track (m)	1	0.0001	0.62%	0.0001	0.0001
Three-Way Interactions	3	0.000533	3.31%	0.000533	0.000178
Days × Ball Speed (mm/s) × Ball Track (m)	3	0.000533	3.31%	0.000533	0.000178
Error	0	0	0	0	0
Total	15	0.016111	100%		

**Table 2:** Factorial contribution analysis of track width from ball-on-disc test.

Source	DF	Seq SS	Contribution	Adj SS	Adj MS
Model	15	800,613	100%	800,613	53,374
Linear	5	689,912	86.17%	689,912	137,982
Days	3	170,606	21.31%	170,606	56,869
Ball Speed (mm/s)	1	19,811	2.47%	19,811	19,811
Ball Track (m)	1	499,496	62.39%	499,496	499,496
Two-Way Interactions	7	92,164	11.51%	92,164	13,166
Days × Ball Speed (mm/s)	3	18,565	2.32%	18,565	6188
Days × Ball Track (m)	3	45,460	5.68%	45,460	15,153
Ball Speed (mm/s) × Ball Track (m)	1	28,140	3.51%	28,140	28,140
Three-Way Interactions	3	18,536	2.32%	18,536	6179
Days × Ball Speed (mm/s) × Ball Track (m)	3	18,536	2.32%	18,536	6179
Error	0	0	0	0	0
Total	15	800,613	100%		

**Table 3:** Factorial contribution analysis of track depth from ball-on-disc test.

Source	DF	Seq SS	Contribution	Adj SS	Adj MS
Model	15	6600.44	100%	6600.44	440.03
Linear	5	4923.31	74.59%	4923.31	984.66
Days	3	2122.69	32.16%	2122.69	707.56
Ball Speed (mm/s)	1	18.06	0.27%	18.06	18.06
Ball Track (m)	1	2782.56	42.16%	2782.56	2782.56
Two-Way Interactions	7	1554.94	23.56%	1554.94	222.13
Days × Ball Speed (mm/s)	3	867.19	13.14%	867.19	289.06
Days × Ball Track (m)	3	354.69	5.37%	354.69	118.23
Ball Speed (mm/s) × Ball Track (m)	1	333.06	5.05%	333.06	333.06
Three-Way Interactions	3	122.19	1.85%	122.19	40.73
Days × Ball Speed (mm/s) × Ball Track (m)	3	122.19	1.85%	122.19	40.73
Error	0	0	0	0	0
Total	15	6600.44	100%		

**Table 4:** Factorial contribution analysis of COF from reciprocating test.

Source	DF	Seq SS	Contribution	Adj SS	Adj MS
Model	15	44.666	100%	44.666	2.978
Linear	5	15.177	33.98%	15.177	3.035
Days	3	9.474	21.21%	9.474	3.158
Frequency (Hz)	1	3.215	7.20%	3.215	3.215
Track (m)	1	2.489	5.57%	2.489	2.489
Two-Way Interactions	7	21.012	47.04%	21.012	3.002
Days × Frequency (Hz)	3	9.094	20.36%	9.094	3.031
Days × Track (m)	3	8.946	20.03%	8.946	2.982
Frequency (Hz) × Track (m)	1	2.972	6.65%	2.972	2.972
Three-Way Interactions	3	8.477	18.98%	8.477	2.826
Days × Frequency (Hz) × Track (m)	3	8.477	18.98%	8.477	2.826
Total	15	44.666	100%		

**Table 5:** Factorial contribution analysis of track width reciprocating test.

Source	DF	Seq SS	Contribution	Adj SS	Adj MS
Model	15	1,402,212	100%	1,402,212	93,481
Linear	5	1,187,163	84.66%	1,187,164	237,433
Days	3	1,090,953	77.80%	1,090,953	363,651
Frequency (Hz)	1	18,090	1.29%	18,090	18,090
Track (m)	1	78,120	5.57%	78,120	78,120
Two-Way Interactions	7	162,223	11.57%	162,223	23,175
Days × Frequency (Hz)	3	122,625	8.75%	122,625	40,875
Days × Track (m)	3	11,037	0.79%	11,037	3679
Frequency (Hz) × Track (m)	1	28,561	2.04%	28,561	28,561
Three-Way Interactions	3	52,826	3.77%	52,826	17,609
Days × Frequency (Hz) × Track (m)	3	52,826	3.77%	52,826	17,609
Total	15	1,402,212	100%		

**Table 6:** Factorial contribution analysis of wear track depth reciprocating test.

Source	DF	Seq SS	Contribution	Adj SS	Adj MS
Model	15	6069.75	100%	6069.75	404.65
Linear	5	3980.5	65.58%	3980.5	796.1
Days	3	2944.25	48.51%	2944.25	981.42
Frequency (Hz)	1	12.25	0.20%	12.25	12.25
Track (m)	1	1024	16.87%	1024	1024
Two-Way Interactions	7	1242.75	20.47%	1242.75	177.54
Days × Frequency (Hz)	3	637.25	10.50%	637.25	212.42
Days × Track (m)	3	461.5	7.60%	461.5	153.83
Frequency (Hz) × Track (m)	1	144	2.37%	144	144
Three-Way Interactions	3	846.5	13.95%	846.5	282.17
Days × Frequency (Hz) × Track (m)	3	846.5	13.95%	846.5	282.17
Total	15	6069.75	100%		

## Data Availability

The datasets presented in this article are not readily available because the data are part of an ongoing study. Requests to access the datasets should be directed to Corresponding Author.

## Abbreviations

The following abbreviations are used in this manuscript:

FRPCs	Fiber-reinforced polymer composites
COF	Coefficient of friction
ANOVA	Analysis of variance
SEM	Scanning electron microscope
